# A novel prognostic model based on migrasome-related LncRNAs for gastric cancer

**DOI:** 10.1038/s41598-025-99781-4

**Published:** 2025-04-25

**Authors:** Wenhao Jiang, Jiaying Shi, Yingchuan Zhu, Lan Yin, Yue Song, Jingfei Zhang, Xinyu Lin, Jiaxiu Zhong, Yilu Lu, Yongxin Ma

**Affiliations:** Department of Medical Genetics, Frontiers Science Center for Disease-related Molecular Network, State Key Laboratory of Biotherapy, West China Hospital, Sichuan University, Cheng Du, 610041 China

**Keywords:** Gastric cancer, Migrasome, Risk prognostic model, Long non-coding RNAs (lncRNAs), LASSO, Cox regression, Cancer, Cancer microenvironment, Cancer screening, Cancer therapy, Gastrointestinal cancer

## Abstract

Gastric cancer (GC) represents a substantial public health challenge, characterized by elevated morbidity and mortality rates. Migrasomes, a newly discovered type of extracellular vesicle, have been highlighted as important contributors to cancer progression, though their specific role in GC remains unclear. To address this issue, we developed the first prognostic model utilizing migrasome-related long non-coding RNAs (MRLs). This model aims to deepen the understanding of GC pathogenesis and improve patient outcomes. Clinical and transcriptional data for 407 GC patients from TCGA were classified as training and testing sets. Through Pearson correlation analysis, 537 MRLs were recognized, and LASSO and Cox regression analyses further refined the list to four key lncRNAs (AC012055.1, LINC01150, AC053503.4, AC107021.2) for constructing the prognostic model. Kaplan-Meier survival analysis indicated a significantly poorer prognosis for the high-risk group. PCA confirmed the model’s robustness, and univariate and multivariate analyses validated it as an independent predictor of clinical outcomes. The ROC curve and C-index evaluations further affirmed the model’s predictive power. We developed a nomogram combining the MRLs signature with clinical parameters to enhance prognostic accuracy. GO, KEGG and GSEA were performed on migrasome-related genes associated with GC. Furthermore, high-risk patients exhibited increased immune cell infiltration and reduced tumor mutation burden, both associated with poorer outcomes. Additionally, twenty-nine potential therapeutic agents were identified. This novel MRLs-based model provides crucial insights into GC biology and represents a valuable tool for improving patient management and therapeutic strategies.

**Supplementary Information:**

The online version contains supplementary material available at 10.1038/s41598-025-99781-4.

## Introduction

Gastric cancer (GC) remains a major global health burden, with over 0.96 million new cases and nearly 0.66 million deaths reported in 2022, making it the fifth leading cause of cancer incidence and mortality across the globe^1^. The prevalence of GC exhibits substantial geographical disparities, with notably higher incidence and mortality rates reported in East Asia, Eastern Europe, and South America^2^. Early-stage GC is frequency asymptomatic or presents with nonspecific symptoms, leading to delayed diagnosis in over 70% of cases and contributing to poor 5-year survival rates^1,3^.

Current therapeutic protocols for GC involve endoscopic screening, followed by surgical intervention and adjuvant chemotherapy (CT) or chemoradiotherapy (CRT). However, surgical eligibility is limited to ~ 50% of patients due to advanced disease progression at diagnosis^2,4,5^. Postoperative recurrence rates exhibit pronounced stage dependence, ranging from 1.19% in stage IA to 73.9% in stage IV disease^6,7^. These challenges underscore the urgent need for improved biomarkers to enable early detection and personalized treatment strategies. While chronic *Helicobacter pylori* infection accounts for ~ 90% of GC cases^8^, tumorigenesis is multifactorial, involving genetic polymorphisms, lifestyle factors (e.g., smoking, alcohol)^9,10^, and dynamic interactions within the tumor microenvironment (TME). Notably, extracellular vesicles such as exosomes have emerged as key mediators of immune evasion and metastatic progression^11^.

Migrasomes, first identified in 2015 as 0.5–3 μm membranous vesicles formed during cell migration^12,13^, facilitate cell-microenvironment crosstalk and promote metastasis^14,15^. Recent research by Cheng et al.^16^ has revealed that anti-migration nanoparticles interact closely with migrasomes and retraction fibers, underscoring their potential as targets for novel anti-metastasis therapies and as valuable sources of cancer biomarkers. However, their specific roles in GC recurrence and progression remain largely unexplored. Moreover, the functional roles of migrasomes in GC recurrence and progression remain unexplored, particularly regarding their cargo of noncoding RNAs. Long non-coding RNAs (lncRNAs, > 200 nucleotides), are crucial epigenetic regulators implicated in GC proliferation, invasion, and drug resistance^17,18^. Despite this, the mechanistic interplay between migrasome-derived LncRNAs (MRLs) and GC pathogenesis is unknown.

To address this this knowledge gap, we systematically identified MRLs and developed a prognostic model utilizing an MRLs matrix. This model demonstrated robust predictive accuracy for survival outcomes and correlated with distinct immune microenvironment, mutation landscape, patient prognosis evaluation, and drug screening. Our findings not only elucidate the role of MRLs in GC pathobiology but also provide a framework for precision oncology approaches targeting migrasome-mediated pathways.

## Materials and methods

### Acquisition of the dates

**Fig. 1:**
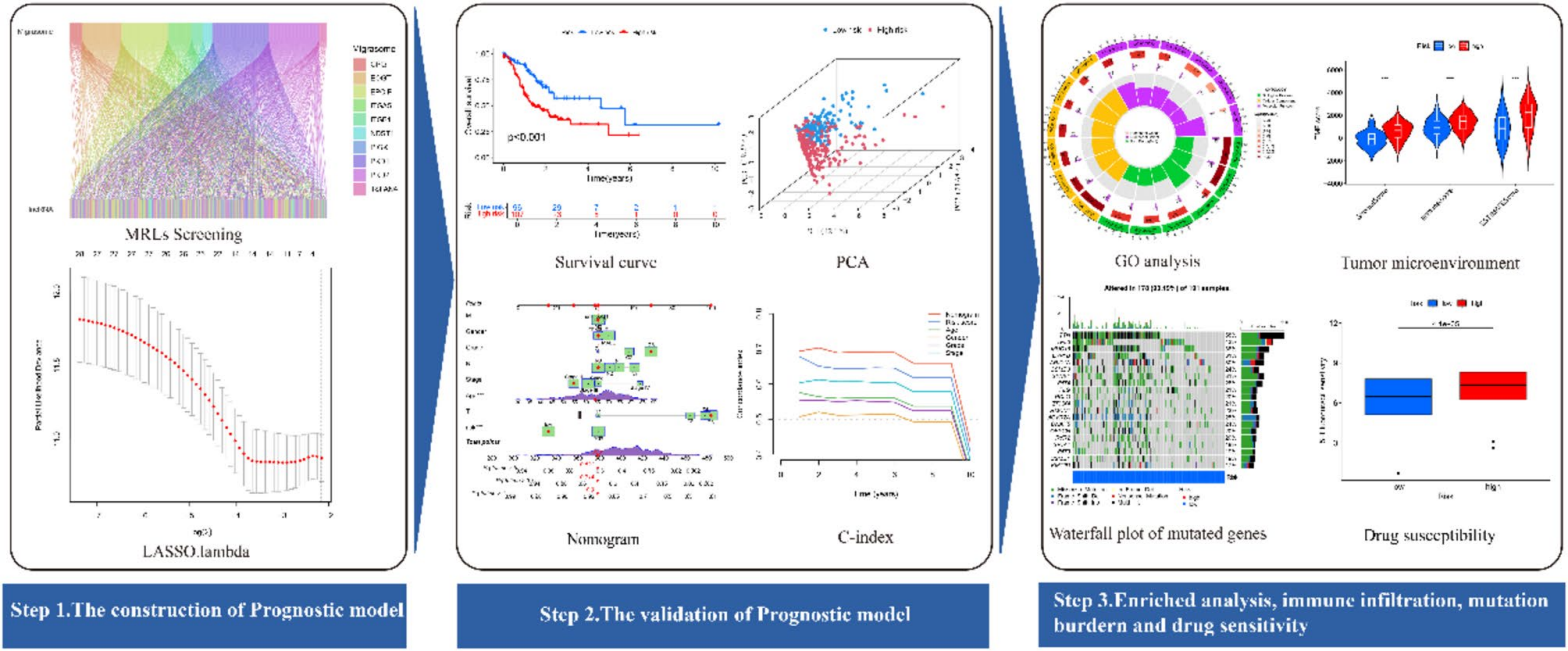
Flowchart depicting the overall study process.

The analytical process undertaken in this study is illustrated in Fig. 1. A total of 448 GC samples, encompassing RNA sequencing (RNA-seq) data, somatic mutation data, and corresponding clinical information, were retrieved from The Cancer Genome Atlas (TCGA) dataset. Samples with incomplete clinical data were excluded from subsequent analyses.

### Screening of migrasome-associated genes and LncRNAs

Through a comprehensive literature review^14,19–23^ and analysis of the GeneCards database^24^ (https://www.genecards.org/, with a relevance score > 1), we identified ten migrasome-related genes (MRGs): *ITGB1*, *ITGA5*, *EOGT*, *CPQ*, *PIGK*, *NDST1*, *TSPAN4*, *EPCIP*, *PKD2*, and *PKD1*.

We then extracted mRNA expression data of MRGs related to GC from TCGA database for further analysis utilizing the limma (v 3.60.4) package in R (v 4.4.1). Co-expression analysis between MRGs and lncRNAs in GC was performed. GC-related MRLs were identified based on a Pearson correlation coefficient (|R|) greater than 0.4 with a p-value less than 0.001.

### Inclusion and exclusion criteria for enrolled patients in the construction of the risk signature

The selection criteria for patients included in the GC model were as follows: (1) a confirmed diagnosis of primary GC; (2) availability of complete clinicopathological data; (3) presence of RNA sequencing data; (4) overall survival (OS) as the primary endpoint; and (5) a minimum follow-up duration of 90 days. Patients were excluded if they had secondary GC, incomplete survival data or missing clinical information.

Study participants were randomly allocated to a training set (N1 = 204) and a test set (N2 = 203). To assess the appropriateness of the randomization, a Chi-square test of independence was used to compare demographic, laboratory, and prognostic data between groups.

### Development of a prediction model using LASSO-Cox regression

Univariate Cox regression analysis was carried out to determine GC-related lncRNAs. Subsequently, the LASSO regression with cross-validation was performed once to refine the selection of variables. LASSO regression minimizes overfitting by shrinking or eliminating coefficients of less relevant genes, setting them to 0, based on the partial likelihood and the lambda (λ) value. The optimal λ value is ascertained through cross-validation by evaluating model performance across a range of λ values, to minimize prediction error or maximize model likelihood. The regularization path, which illustrates how gene coefficients change as λ varies, helps to identify which genes are retained (i.e., have non-zero coefficients) at a given λ value^25^. The LASSO risk score was determined by applying the following formula:

$$\:\varvec{L}\varvec{A}\varvec{S}\varvec{S}\varvec{O}\:\varvec{r}\varvec{i}\varvec{s}\varvec{k}={\sum\:}_{\varvec{i}=1}^{\varvec{n}}\varvec{C}\varvec{o}\varvec{e}\varvec{f}\varvec{i}\:\times\:\:\varvec{x}\varvec{i}$$,

where Coefi denotes the coefficient of the i-th gene, and xi denotes the corresponding gene expression level.

The R packages survival (v 3.7.0), survminer (v 0.4.9), and glmnet (v 4.1.8) were used for the analyses in this section. The R package timeROC (v 0.4) was utilized to calculate the area under the curve (AUC) for the prediction model.

### Evaluation of the constructed risk model

The model’s predictive accuracy was evaluated utilizing risk curve analysis, Kaplan-Meier (K-M) survival analysis, operating characteristic (ROC) curve analysis, principal component analysis (PCA)^26^ and independent prognostic analysis for the entire cohort, as well as for the training and testing sets, respectively.

The maximum AUC value, representing the model’s optimal discriminatory ability, was determined by evaluating the entire ROC curve. Based on this maximum AUC value, the model was identified as the best candidate among the compared models. The 1-, 3-, and 5-year ROC curves were plotted. To validate the cut-off point based on the MRLs risk score, we conducted a K-M analysis on the risk prognostic models.

### Development and validation of a nomogram

Univariate and multivariate Cox regression analyses were conducted to pinpoint the relevant variables for constructing the nomogram. Utilizing the survival package (v 3.7.0) in R, a forest plot was generated. Molecular markers related to GC prognosis (MRLs) were recognized as independent prognostic factors for predicting survival in the TCGA-STAD dataset.

The nomogram was devised to provide clinical prognostications for GC patients by integrating the risk scores with other clinicopathological attributes. Calibration curve and decision curve analysis (DCA) were performed to evaluate the clinical reliability and utility of the nomogram.

### Identification and functional enrichment of DEGs

To gain a more profound understanding of the potential cellular functions and pathways, we identified differentially expressed genes (DEGs) between the high- and low-risk groups utilizing the limma package (v 3.60.4) in R. The criteria for DEG selection were set to an absolute |log2 fold change (FC)| > 2 and an adjusted P-value < 0.01. Functional enrichment analyses were conducted by the clusterProfiler package (v 4.12.6)^27^. These analyses included Gene Ontology (GO)^28^, Kyoto Encyclopedia of Genes and Genomes (KEGG)^29–31^, Gene Set Enrichment Analysis (GSEA)^32^ analysis.

### Immune microenvironment analysis and cell infiltration estimation

The stromal score, immune score, ESTIMATE score, and tumor purity were calculated by applying the ESTIMATE algorithm via estimate package (v1.0.13)^33^. To further quantify the relative abundance of infiltrating immune cells within the TME, the CIBERSORT algorithm^34^ was utilized to analyze the RNA-Seq data of GC patients, assessing 22 distinct immune cell types^34^. To further quantify immune cell infiltration in each sample, single-sample Gene Set Enrichment Analysis (ssGSEA) was performed to evaluate the enrichment of 29 immune signatures^35^, using the GSVA package (v1.52.3)^36^. The Pearson correlation coefficient was utilized to evaluate the association between the risk score and immune cell infiltration. The potential response to immunotherapy was assessed using the Tumor Immune Dysfunction and Exclusion (TIDE) (http://tide.dfci.harvard.edu) score^37^.

### Drug sensitivity analysis

To identify potential therapeutic agents with increased efficacy in high-risk GC patients, a drug sensitivity analysis was conducted. The predictive model was trained using cell line data obtained by the Genomics of Drug Sensitivity in Cancer (GDSC, https://www.cancerrxgene.org/)^38^. The oncoPredict (version 1.2) in R was used to determine chemotherapeutic sensitivity within the TCGA-STAD datasets^39^.

## Results

### Identification of the migrasome-related signature

Patients were randomly assigned to a training set for model construction (Table S3) and a testing set for evaluation (Table S4). A Chi-square test of independence revealed no notable differences in any of the variables between the two groups, confirming the randomness and effectiveness of the grouping (Table 1).

**Table 1:** Basal clinicopathologic characteristics in training set and test set.

Covariates	Type	Total *N* = 407	Training N1 = 204	Test N2 = 203	*P*-value
Age	<=65	183(44.96%)	90(44.12%)	93(45.81%)	0.8416
Age	> 65	221(54.3%)	112(54.9%)	109(53.69%)	
Age	Unknown	3(0.74%)	2(0.98%)	1(0.49%)	
Gender	Female	144(35.38%)	70(34.31%)	74(36.45%)	0.7281
Gender	Male	263(64.62%)	134(65.69%)	129(63.55%)	
Grade	G1	12(2.95%)	5(2.45%)	7(3.45%)	0.5581
Grade	G2	144(35.38%)	77(37.75%)	67(33%)	
Grade	G3	242(59.46%)	118(57.84%)	124(61.08%)	
Grade	Unknown	9(2.21%)	4(1.96%)	5(2.46%)	
Stage	Stage I	55(13.51%)	29(14.22%)	26(12.81%)	0.7812
Stage	Stage II	122(29.98%)	59(28.92%)	63(31.03%)	
Stage	Stage III	167(41.03%)	86(42.16%)	81(39.9%)	
Stage	Stage IV	39(9.58%)	17(8.33%)	22(10.84%)	
Stage	Unknown	24(5.9%)	13(6.37%)	11(5.42%)	
T	T1	21(5.16%)	14(6.86%)	7(3.45%)	0.3347
T	T2	86(21.13%)	41(20.1%)	45(22.17%)	
T	T3	179(43.98%)	84(41.18%)	95(46.8%)	
T	T4	113(27.76%)	59(28.92%)	54(26.6%)	
T	Unknown	8(1.97%)	6(2.94%)	2(0.99%)	
M	M0	362(88.94%)	185(90.69%)	177(87.19%)	0.4467
M	M1	26(6.39%)	10(4.9%)	16(7.88%)	
M	Unknown	19(4.67%)	9(4.41%)	10(4.93%)	
N	N0	121(29.73%)	57(27.94%)	64(31.53%)	0.2844
N	N1	108(26.54%)	58(28.43%)	50(24.63%)	
N	N2	77(18.92%)	43(21.08%)	34(16.75%)	
N	N3	82(20.15%)	35(17.16%)	47(23.15%)	
N	Unknown	19(4.67%)	11(5.39%)	8(3.94%)	

**Fig. 2:**
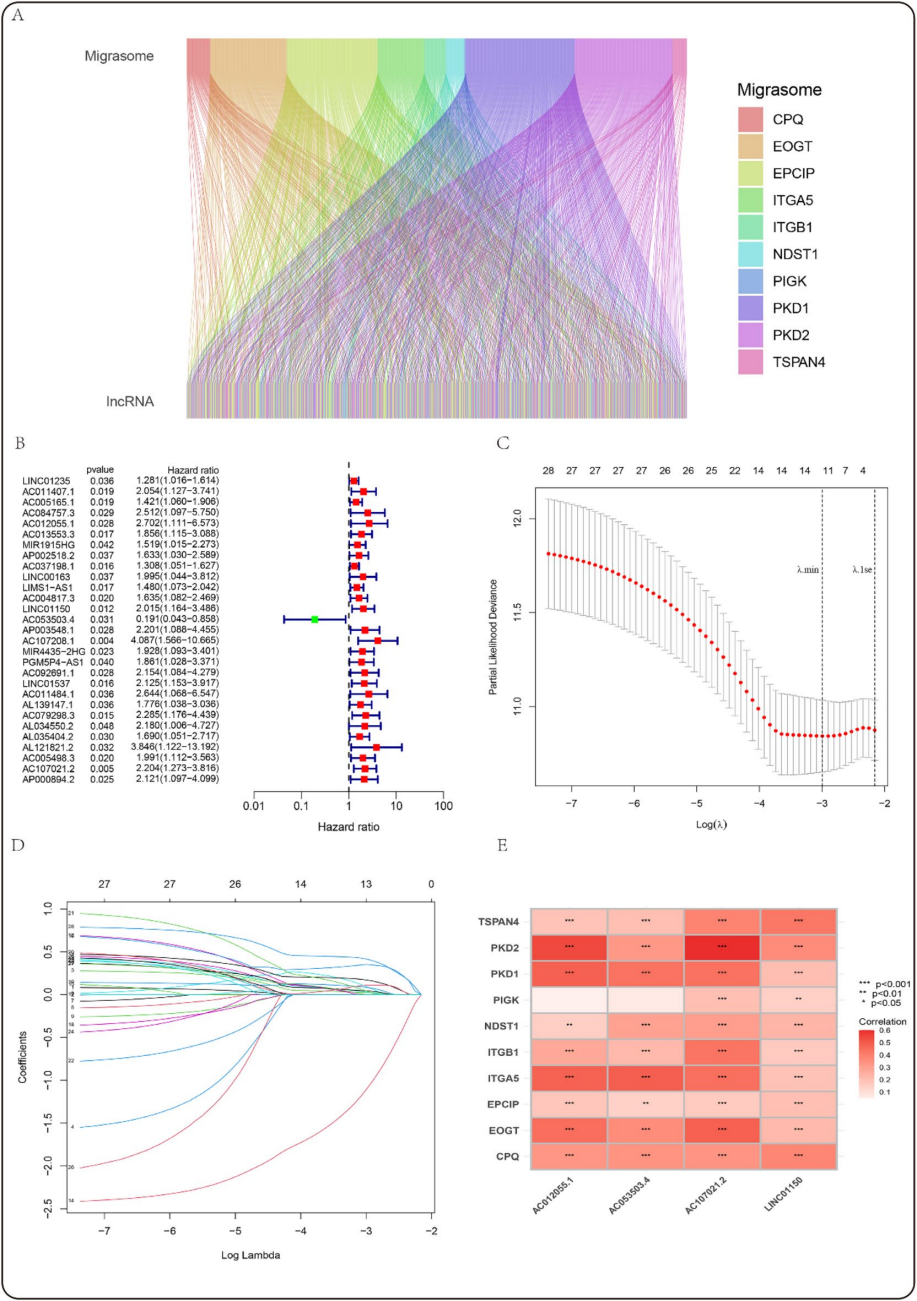
Development of a prognostic model for MRLs in gastric cancer (GC). (**A**) Pearson correlation analysis of Migrasome gene co-expression in GC, obtained from the lncRNA Sanger map; (**B**) Univariate regression of MRLs in GC; (**C**, **D**) LASSO regression of MRLs in GC. In panel (C) Tenfold cross-validation error rate plots, the two dashed lines represent two specific λ values: λ.min and λ.1se. (**D**) demonstrates the significance of each variable, where distinct variables are represented by lines of different colors. As the penalty term (λ) increases, the variables are increasingly penalized, resulting in alterations to their coefficients; (**E**) Clustering heatmap showing the expression of 10 MRGs and 4 MRLs.

A comprehensive co-expression analysis was performed to detect lncRNAs associated with the migrasomes in GC. A total of 537 lncRNAs corresponding to 1,202 lncRNA-migrasome pairs were identified (Fig. 2A, table S2). Then, a univariate Cox regression was performed to ascertain MRLs linked to OS in GC. The results indicate that migrasomes exert a significant and intricate role in the pathogenesis of GC. The analysis identified 28 MRLs as risk factors, including LINC01235, AC011407.1, AC005165.1, AC084757.3, AC012055.1, AC013553.3, MIR1915HG, AP002518.2, AC037198.1, LINC00163, LIMS1-AS1, AC004817.3, LINC01150, AP003548.1, AC107208.1, MIR4435-2HG, PGM5P4-AS1, AC09269.1, LINC01537, AC011484.1, AL139147.1, AC079298.3, AL034550.2, AL035404.2, AL121821.2, AC005498.3, AC107021.2, and AP000894.2, with hazard ratios ranging from 1.281 to 4.087. Additionally, one protective factor, AC053503.4, was determined to have a hazard ratio of 0.191 (Fig. 2B). Subsequently, LASSO regression analysis was employed to further refine the candidate MRLs, resulting in the identification of four MRLs with optimal λ values (Fig. 2C,D). Based on these findings, a risk formula was developed, incorporating the expression levels of the selected MRLs as described below: Risk Score = (0.763 * expression of AC012055.1) + (0.472 * expression of LINC01150) + (-2.168 * expression of AC053503.4) + (0.851 * expression of AC107021.2) (Fig. 2E).

### Assessment and verification of the prognostic model

**Fig. 3:**
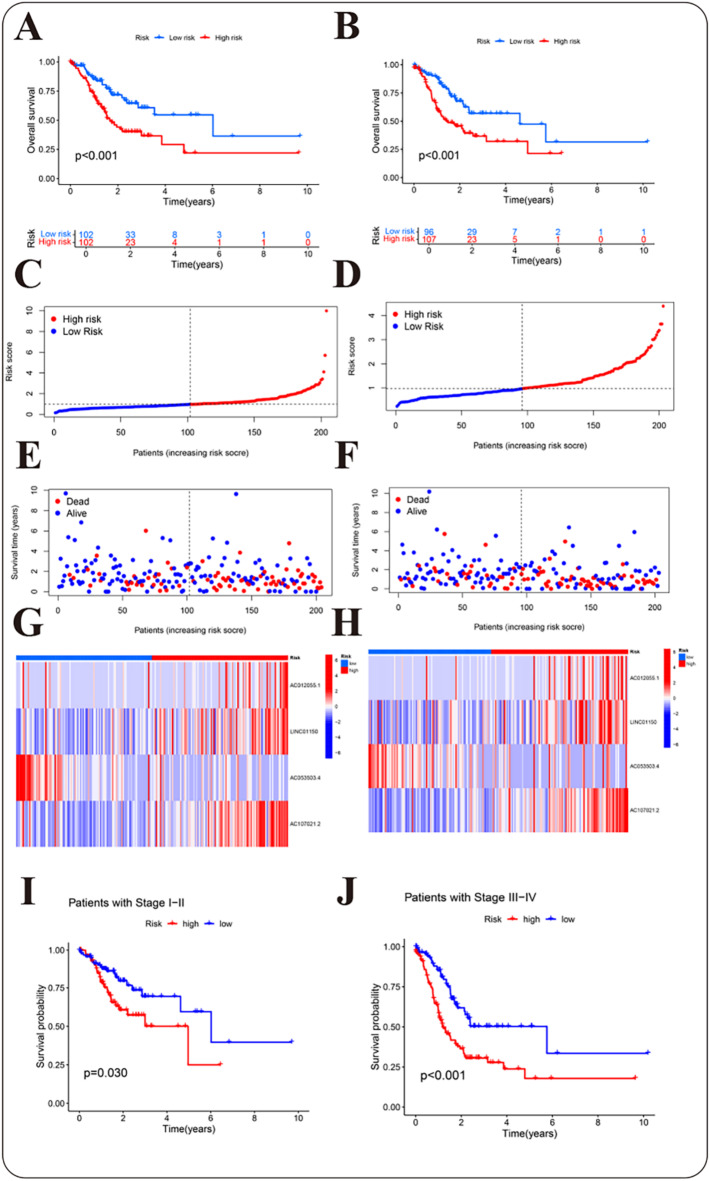
Categorization and evaluation of risk prediction models. (**A**) K-M survival curve of the training subset patients. (**B**) K-M survival curve of the test subset patients. (**C**) Scatter plot representing risk scores of the training subset. (**D**) Scatter plot representing risk scores of test subset. (**E**) Scatter plot illustrating survival status of the training subset patients. (**F**) Scatter plot depicting survival status of the test subset patients. (**G**) Cluster analysis plot for the training subset patients. (**H**) Cluster analysis plot for the test subset patients. (**I**) Survival curves for early-stage (I-II) GC patients. (**J**) Survival curve for advanced-stage (III-IV) GC patients. In subfigures (C) to (H), the dashed line in the center represents the median value, which serves as the threshold to differentiate between different risk individuals. Patients positioned to the left of the median are classified as low risk (shown in blue), whereas those to the right are classified as high risk (shown in red).

Patients were stratified into different risk groups based on the median risk score derived from the formula, resulting in an equal distribution with a 1:1 ratio. By comparing the distribution of risk scores, we observed notable disparities in OS rates and gene expression profiles of the four long non-coding RNAs included in the prognostic model throughout the entire TCGA dataset (Fig. S1).These results are further stratified into training (Fig. 3A,C,E,G) and test sets (Fig. 3B,D,F,H), demonstrating consistent patterns in both cohorts. The results indicated that patients with higher risk scores exhibited notably poorer survival rates and correspondingly higher mortality rates. K-M survival was developed on both the training and testing subsets. The results suggested that patients classified as low-risk exhibited markedly improved outcomes than those classified as high-risk group, with *p*-values < 0.001 noted in both subsets (Fig. 3A,B). Regardless of early- phase (I-II) or advanced- phase (III-IV), patients classified as high-risk group showed notably decreased survival times than individuals classified as low-risk group (Fig. 3I,J).

**Fig. 4:**
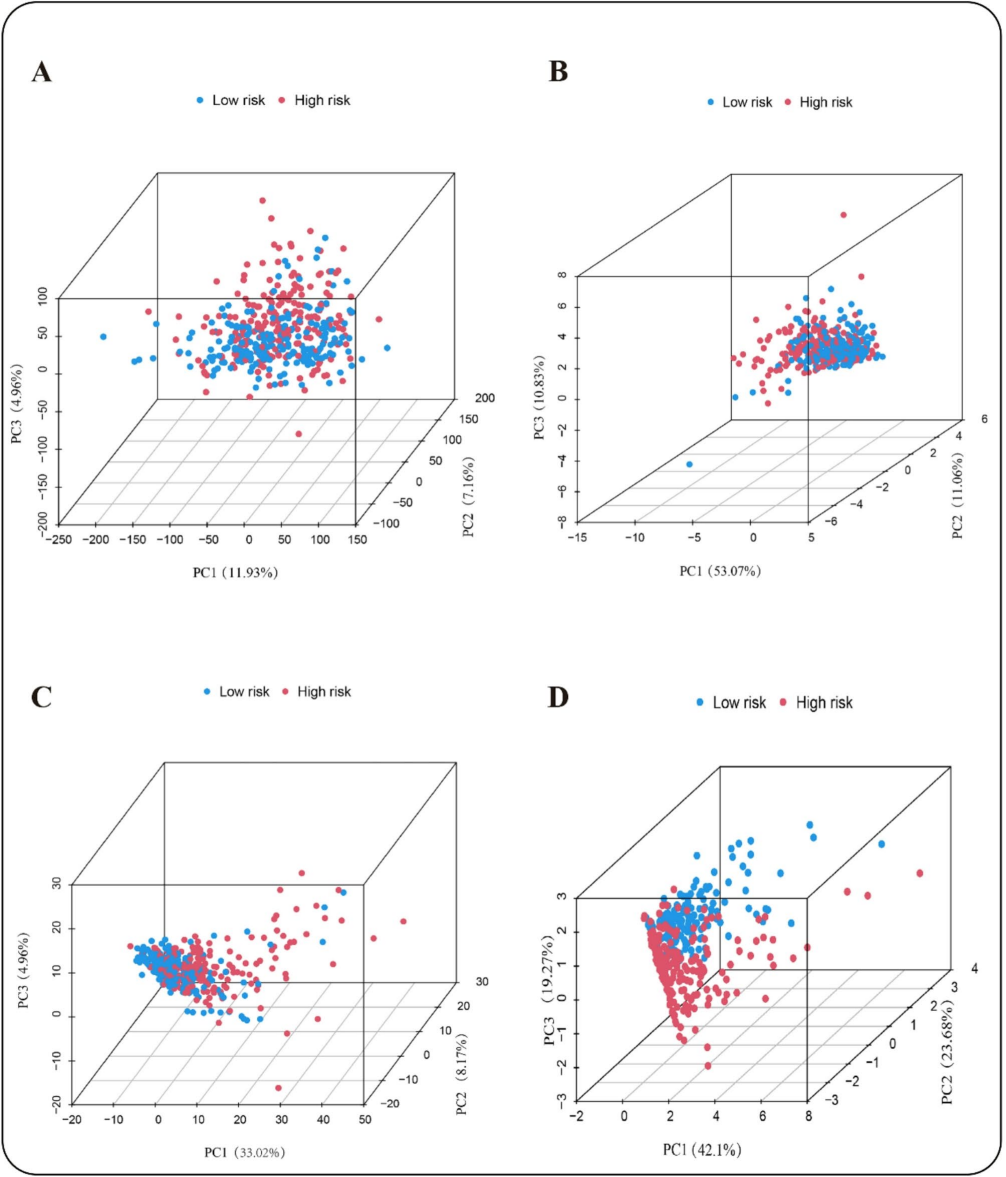
Principal component analysis of risk prognostic models. (**A**) PCA conducted on the complete gene set demonstrates the capacity to differentiate between different risk individuals. The first three principal components accounted for 11.9%, 7.16%, and 4.76% of the variance, respectively. (**B**) PCA focused on MRGs reveals the capacity to differentiate high-risk from low-risk populations, with the top three components explaining 53.07%, 11.6%, and 10.83% of the variance.(**C**) PCA analysis of MRLs indicates their efficacy in differentiating between different risk individuals, with the top two components accounting for 33.02%, 8.07%, and 4.96% of the variance. (**D**) PCA of the risk model effectively differentiates between different risk groups, with the top three components (from four lncRNAs) accounting for 42.1%, 23.68%, and 19.27% of variance.

To further validate the efficacy of the new constructed prognostic model, we assessed the discriminatory power of four distinct gene sets using PCA: the complete gene set, migrasome-related genes (MRGs), MRLs, and the long non-coding RNAs incorporated in the model. The PCA results revealed that the first three principal components accounted for 11.9%, 7.16%, and 4.76% of the total variance (23.82%) in differentiating between high- and low-risk individuals when considering all genes. In contrast, for MRGs, these components explained 53.07%, 11.6%, and 10.83% of the variance, respectively, yielding a cumulative variance of 75.5%. For MRLs, the proportions were 33.02%, 8.07%, and 4.96%, contributing to a total variance of 46.05%. Notably, the PCA derived from our constructed risk prognostic model demonstrated a strong ability to differentiate between different risk individuals, with the first three components accounting for 42.1%, 23.68%, and 19.27% of the variance, respectively, resulting in a cumulative variance of 85.05%. These findings indicate that our risk model exhibited the highest variance contribution, effectively encapsulated population variance and demonstrated a significant ability to differentiate between patients categorized into different risk cohorts. In summary, while the initial three gene sets exhibited limited classification capability, the proposed risk diagnostic model accurately distinguished between different risk individuals (Fig. 4A–D).

**Fig. 5:**
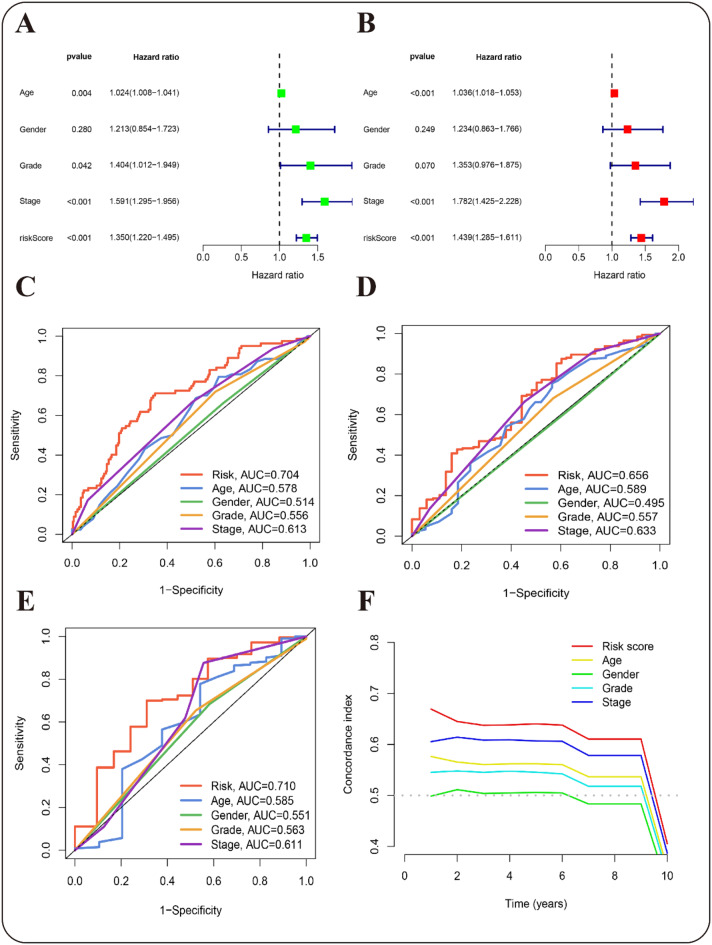
Assessment of the predictive performance of prognostic risk Models employing MRLs in GC. (**A**) Univariate regression. (**B**) Multivariate regression. (**C**–**E**) ROC curve analysis: ROC curves depict the balance between sensitivity and specificity for predicting 1-year (C), 3-year (D), and 5-year (E) survival across different variables. (**F**) C-index Validation: The C-index assesses the model’s ability to correctly predict the order of event occurrence among randomly paired patients. C-index of 0.5 (dotted line) signifies no predictive power; values exceeding this indicate predictive efficacy.

To assess the independence and predictive performance of the newly constructed prognostic model, we conducted univariate and multivariate regression analyses, along with ROC curve and C-index assessments. In the univariate analysis, the risk model exhibited a hazard ratio (HR) of 1.305 (95% CI: 1.22–1.495, *p* < 0.001), suggesting that the model could potentially act as an independent prognostic factor (Fig. 5A, S5A,C). This finding was supported by the multivariate analysis, which revealed that the risk model persisted as an independent predictor of clinical outcomes among GC patients (HR: 1.439, 95% CI: 1.285–1.611, *p* < 0.001) (Fig. 5B, S5B,D). ROC analysis confirmed the model’s independence from other clinical factors, with AUC values of 0.704 for 1-year, 0.656 for 3-year, and 0.710 for 5-year survival in GC patients. Its predictive performance was comparable to that of the earlier gastric cancer prediction model^40,41^. These findings indicate that the model’s predictive ability remained consistent across different time points and subsets (Fig. 5C–E, Fig. S3). Additionally, the C-index analysis verified that the MRL-based risk prognostic model operated independently of other clinical factors (Fig. 5F).

### Construction of nomogram

**Fig. 6:**
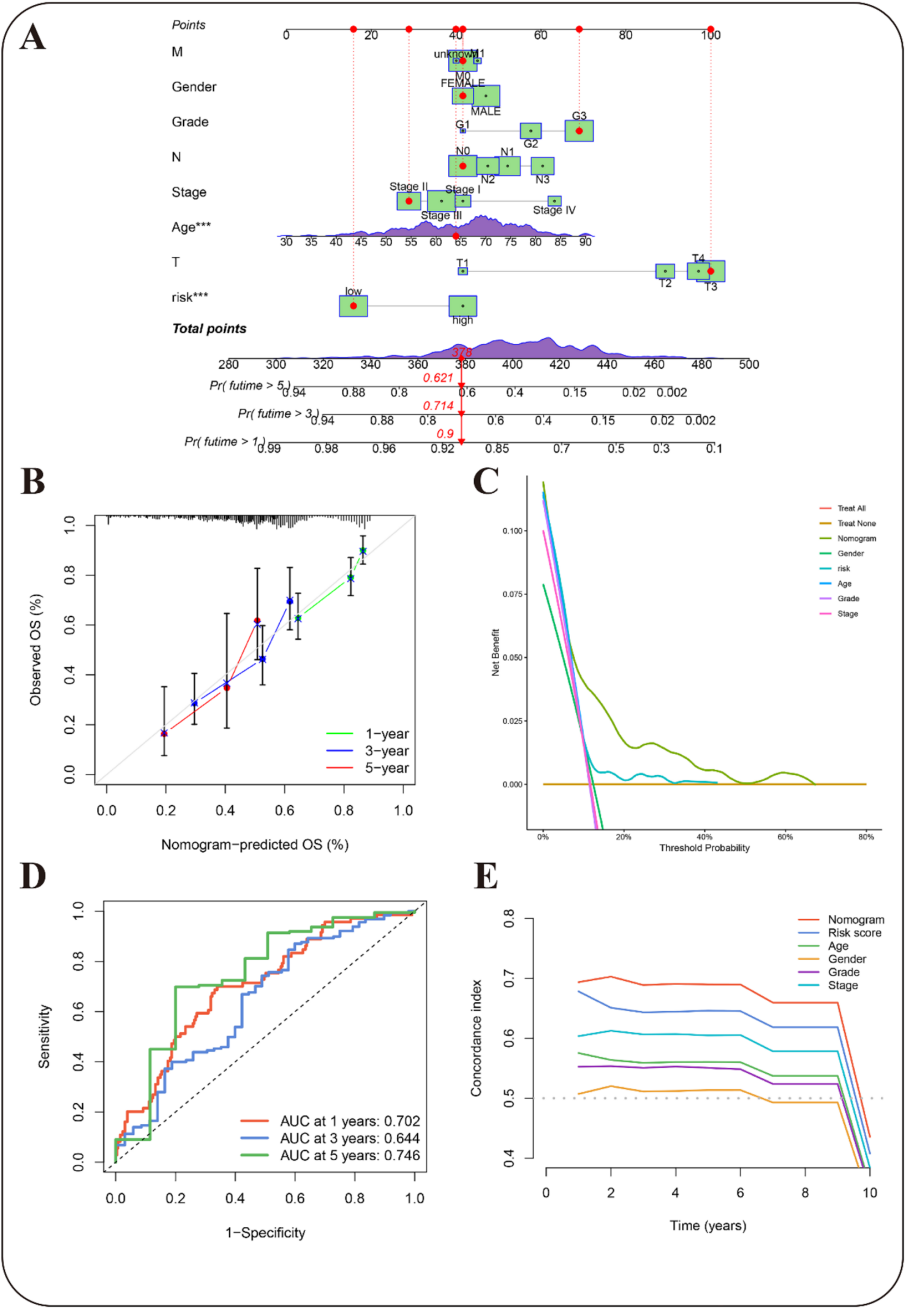
Verification of the predictive model and construction of a nomogram encompassing clinical features. (**A**) The nomogram predicts OS rates for GC patients. Red lines/dots assign points to variables; total points (378) indicate probabilities: 90% for 1-year, 71.4% for 3-year, and 62.1% for 5-year OS. (**B**) Calibration curve assesses the precision of the nomogram model for forecasting 1-, 3-, and 5-year survival rates. (**C**) DCA illustrates the overall improvement of the predictive risk model. (**D**) ROC curves show the model’s predictive accuracy. (**E**) C-index results validate the model’s predictive performance.

To create a more practical tool for individualized prognosis prediction, a nomogram was developed incorporating the risk score, age, clinical stage, gender, M stage, T stage and N stage (Fig. 6A). The final nomogram scores, calculated by integrating all variables, were used to predict 1-, 3-, and 5-year OS rates for each patient. The GC patient under examination exhibited predicted probabilities of 0.9, 0.714, and 0.621 for 1-, 3-, and 5-year OS, respectively.

Calibration curves were generated to verify the alignment between the nomogram’s predictive outputs and actual patient outcomes, revealing a robust concordance between the clinical results and the predicted values (Fig. 6B). Furthermore, DCA was performed to assess the model’s performance, which disclosed a higher net benefit associated with the risk score (Fig. 6C). The ROC curves during the 1-, 3-, and 5-year follow-up intervals yielded AUC values of 0.702, 0.644, and 0.746, respectively, indicating superior predictive performance compared to other clinical indicators (Figs. 5C,D and 6D). The model’s precision was further confirmed by the C-index (Fig. 6E), affirming its potent predictive capacity.

In conclusion, these findings underscore that the nomogram, which amalgamates the migrasome predictive signature with clinical attributes, effectively predicts the clinical prognosis of GC patients.

### Functional enrichment

**Fig. 7:**
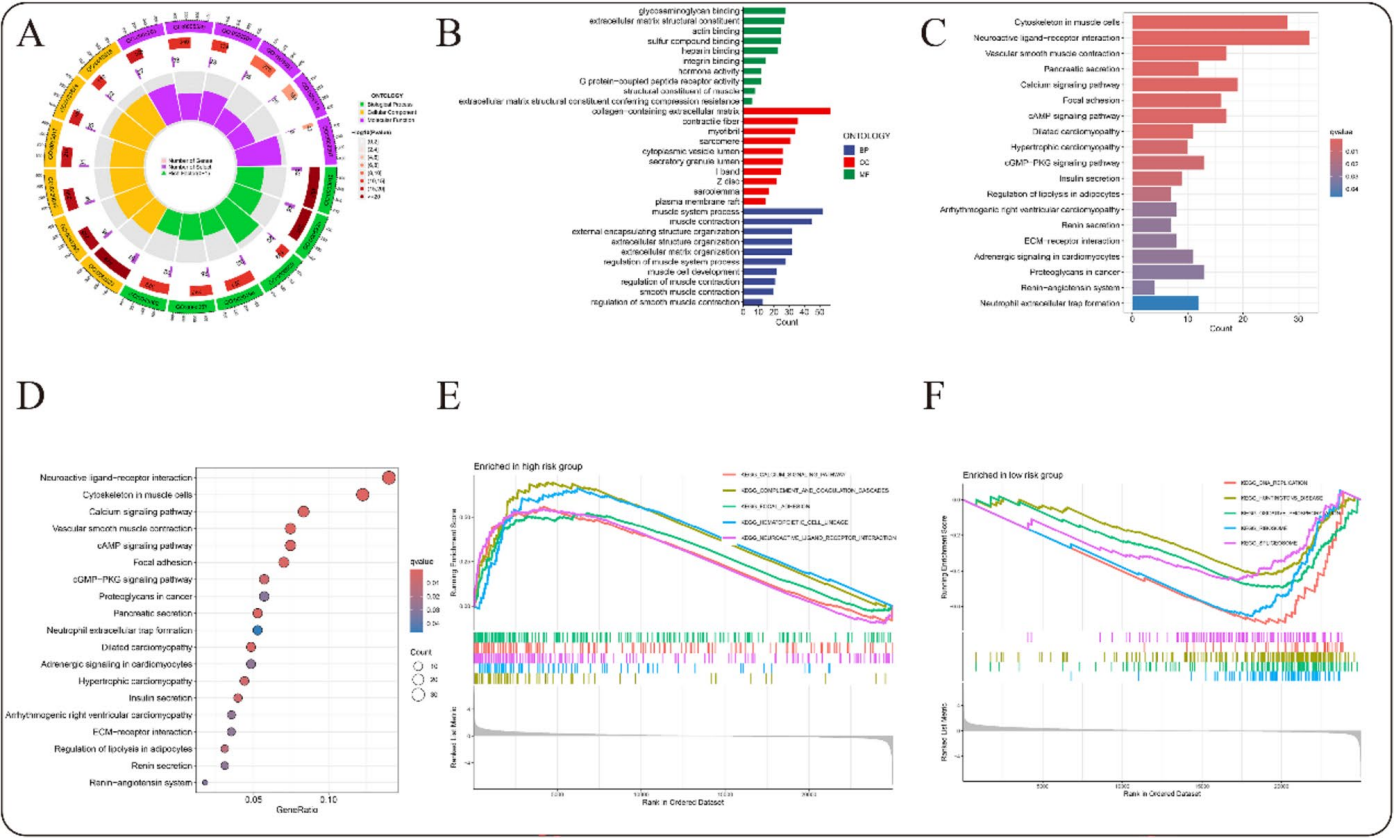
Signaling pathways enriched. (**A**) Circular graph depicting the results of GO analysis. (**B**) Bar graph illustrates the GO. (**C**) Bubble diagram representing the KEGG results. (**D**) Bar graph showing the KEGG results. (**E**) GSEA results of the high-risk group. (**F**) GSEA results of the low-risk group.

We conducted GO among DEGs across different risk populations (Table S7), identifying significant associations in biological processes (BP), cellular components (CC), and molecular functions (MF). Notably, top BP enrichments pertained to muscle system process, muscle contraction, and external structure organization (Figs. 7A,B, S6A,B, S7A,B, table S8). Regarding CC, enrichments centered around collagen-containing extracellular matrix, contractile fiber, and myofibril (Figs. 7A,B, S6A,B, S7A,B, table S8). For MF, the most enriched terms were glycosaminoglycan binding, extracellular matrix structural constituent, and actin binding (Figs. 7A,B, S6A,B, S7A,B, table S8). KEGG pathway analysis identified the top five enriched pathways as cytoskeleton in muscle cells, neuroactive ligand-receptor interaction, vascular smooth muscle contraction, pancreatic secretion and calcium signaling pathway (Figs. 7C,D, S6C,D, S7C,D, table S9). GSEA identified distinct molecular functions between the two GC subtypes. In the high-risk GC group, the five most significantly enriched pathways included neuroactive ligand-receptor interaction, hematopoietic cell lineage, focal adhesion, complement and coagulation cascades, and the calcium signaling pathway (Figs. 7E, S6E, S7E, table S10). Conversely, in the low-risk GC group, the top five enriched pathways were spliceosome, ribosome, oxidative phosphorylation, Huntington’s disease, and DNA replication (Figs. 7F, S6F, S7F, table S10).

### Identification of the immune landscape

**Fig. 8:**
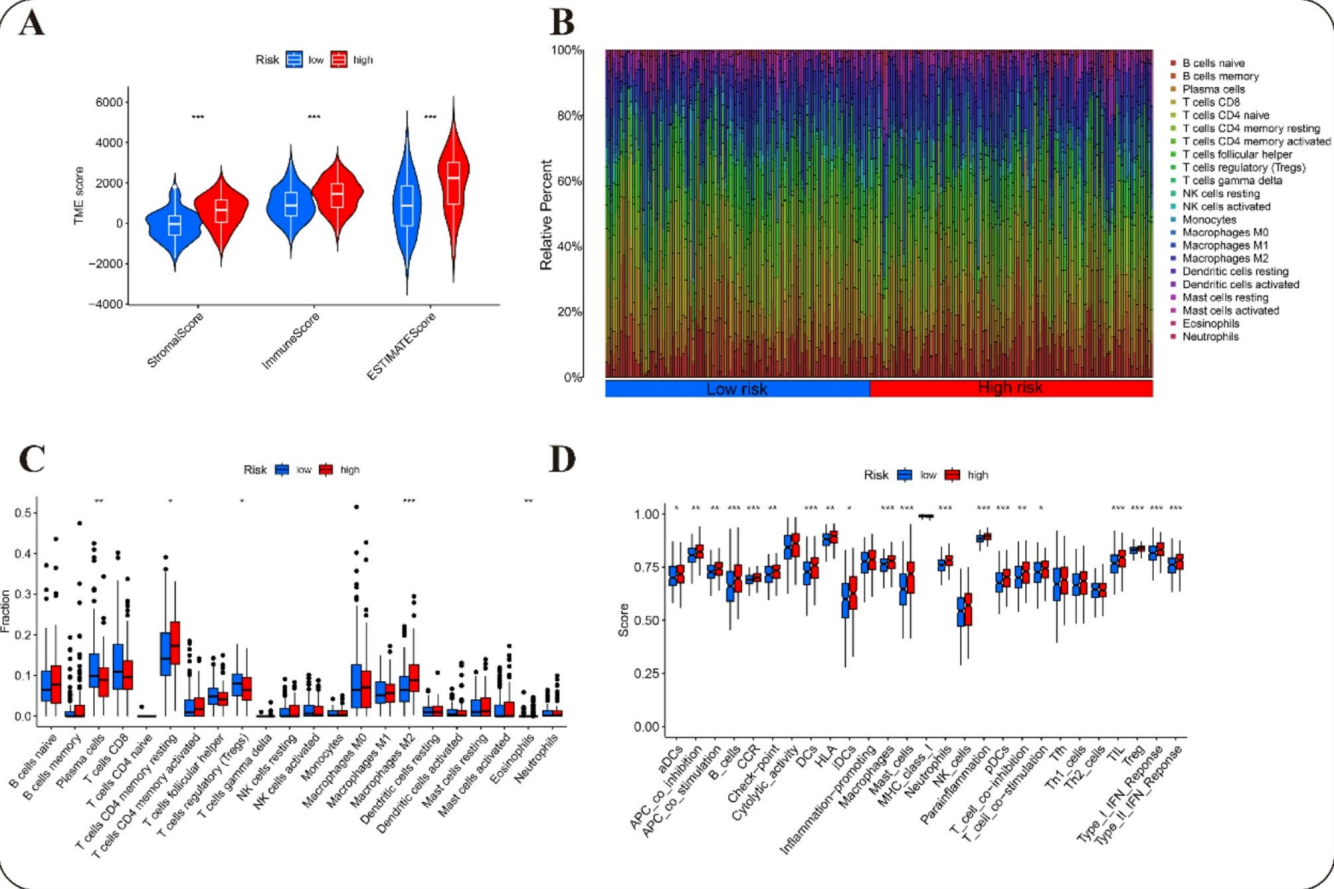
Immunological profile analysis of the prognostic model. (**A**) Tumor microenvironment analysis. (**B**) Immune cell percentage distribution. (**C**) Immune score analysis for both groups. (**D**) immunofunctional analysis. *p < 0.05, **p < 0.01, ***p < 0.001.

First, we analyzed and compared the composition of immune cells between different risk subgroups. The results indicated that the high-risk subgroup exhibited notably elevated TME scores, indicating a more altered TME, and exhibited lower tumor purity than the low-risk subgroup (Figs. 8A, S8A, S9A). Furthermore, the proportion of immune cells was significantly greater in the high-risk subgroup, which correlated with a poorer prognosis (Figs. 8B, S8B, S9B).

Next, utilizing the CIBERSORT algorithm, we estimated the proportions of 22 distinct immune cell types from GC samples retrieved from the TCGA. The analysis demonstrated that patients in the high-risk subgroup exhibited significantly higher proportions of CD4 + memory resting T cells, M2 macrophages, and eosinophils, while plasma cells and regulatory T cells were more prevalent in the low-risk subgroup (*p* < 0.05) (Figs. 8C, S8C, S9C, table S6).

Finally, ssGSEA was employed to assess the levels of various immune cell types and immune functions in different risk groups. The high-risk group exhibited significantly elevated levels of ten distinct immune cell types, such as activated dendritic cells (aDCs), B cells, dendritic cells (DCs), among others. (*p* < 0.05). Additionally, ten immune functions were more active in the high-risk group, suggesting a more robust immune response (Figs. 8D, S8D, S9D, table S11) (*p* < 0.05). These findings highlight notable disparities in tumor immune infiltration between different risk subgroups.

### Differential mutational profiles

**Fig. 9:**
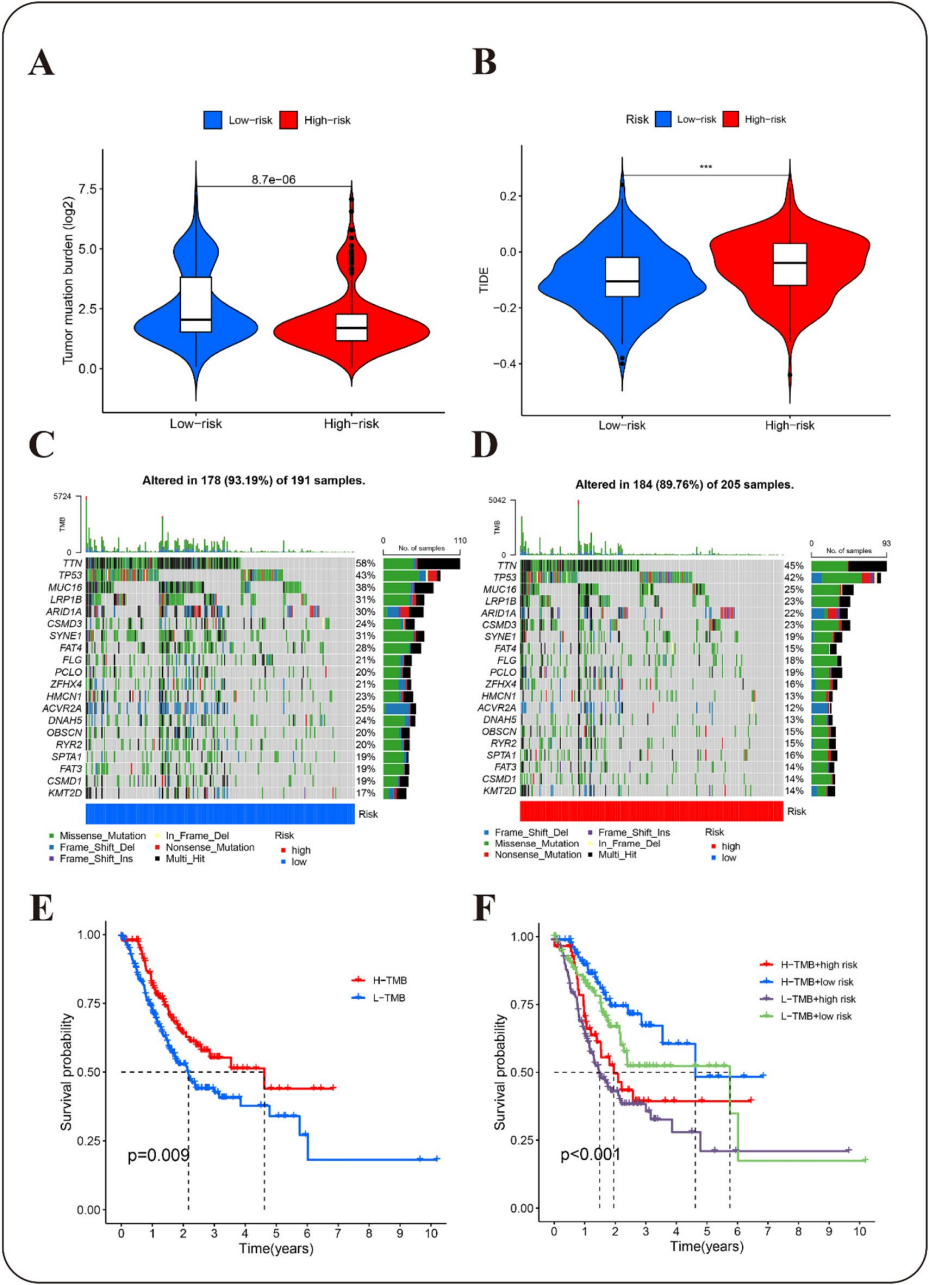
Analysis of immunosignatures in prognostic models. (**A**) TMB scoring. (**B**) TIDE analysis. (**C**) Waterfall plot depicting the 20 most frequently mutated genes in the low-risk subgroup. (**D**) Waterfall plot illustrates the 20 most frequently mutated genes in the high-risk subgroup. (**E**) K-M survival analysis of the two groups with high and low TMB scores. (**F**) K-M survival analysis of four subgroups categorized by TMB and risk status. ***p < 0.01.

To further explore the immunological features of the various risk groups, we performed a thorough analysis of gene mutations. The tumor mutation burden (TMB) analysis indicated that the low-risk group exhibited a notably elevated TMB score than the high-risk group (Figs. 9A, S10A, S11A, table S5). Additionally, TIDE analysis, a widely utilized tool for evaluating tumor immune escape, indicated that the high-risk population might gain greater advantage from immune therapy (Figs. 9B, S10B, S11B, table S14).

We further investigated gene mutations between different risk subgroups by analyzing and visualizing the 20 most frequently mutated genes within the TCGA cohort. The low-risk group exhibited an increased mutation rate (Figs. 9C, S10C, S11C, table S12) than the high-risk group (Figs. 9D, S10D, S11D, table S13), with missense mutations being the predominant mutation type. TTN, TP53, and MUC16 were identified as the genes exhibiting the highest frequency of mutations, each with mutation frequencies exceeding 25% in both subgroups.

Moreover, previous studies across various tumor types have demonstrated that GC patients with a high TMB generally exhibit improved survival outcomes^42,43^. Consistent with these findings, our results indicate that patients in the high TMB (H-TMB) group experienced significantly extended survival times than those in the low TMB (L-TMB) group (*p* < 0.01) (Figs. 9E, S10E, S11E). Further K-M survival analysis indicated that the low-risk + H-TMB group had the longest survival time, whereas the high-risk + L-TMB group had the shortest survival time (*p* < 0.01) (Figs. 9F, S10F, S11F, table S15).

### Drug sensitivity analysis

**Fig. 10:**
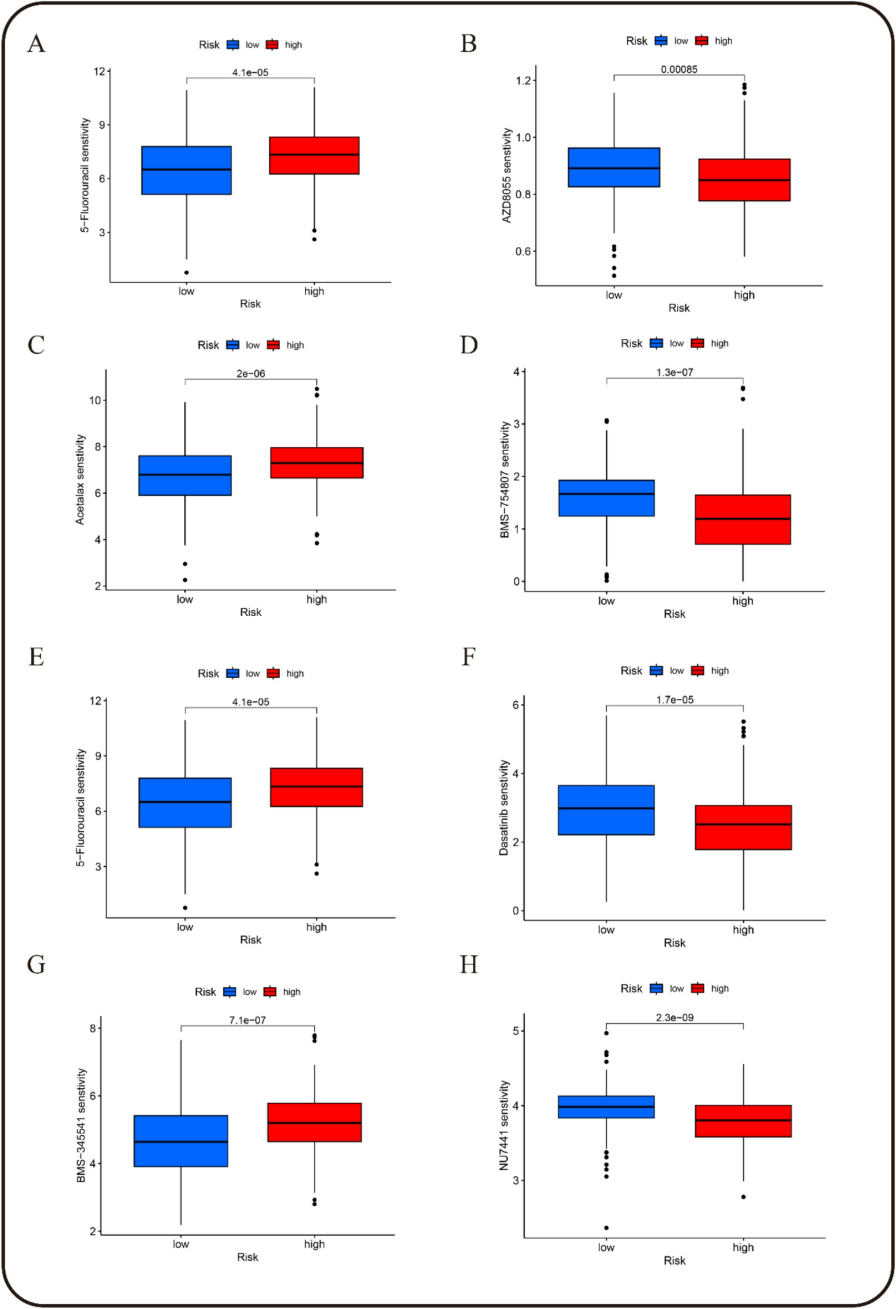
Analysis of drug sensitivity in the risk prognostic model.

To predict drug sensitivity, a drug sensitivity analysis was performed for different risk GC groups. The results indicated that the low-risk GC group showed higher sensitivity to acetalax, AZD8055, BMS-754,807, dasatinib, JQ1, NU7441, palbociclib, and SB216763, with p-values of 2e-06, 8.5e-04, 1.3e-07, 1.7e-05, 1.9e-11, 2.3e-09, 5.9e-06, and 2.6e-05, respectively. In contrast, the low-risk group displayed lower sensitivity to 5-fluorouracil (5-FU), afatinib, AZD3759, BMS-345,541, dabrafenib, dihydrorotenone, gefitinib, GSK1904529A, lapatinib, MK1775, ML323, OSI027, oxaliplatin, pevonedistat, SCH772984, TAF1, ulixertinib, VE821, VE822, and VX-112, with p-values of 4.1e-05, 1.4e-05, 1.1e-04, 7.1e-07, 3.6e-06, 3.7e-04, 1.5e-04, 1.3e-04, 6.1e-08, 6.1e-04, 6.8e-06, 2.1e-11, 1.1e-06, 5e-06, 7.5e-04, 3.3e-04, 6.8e-07, 5.8e-04, 8.9e-05, and 2.2e-05, respectively (Figs. 10, S12–14). These findings suggest distinct drug response profiles between low-risk and high-risk GC groups, which could inform targeted therapeutic strategies.

## Discussion

Gastric cancer, a leading cause of lobal cancer mortality, poses a significant threat to human health and impedes social development^1–3^. While surgical resection and adjuvant therapies offer curative potential for early-stage disease, over 70% of patients progress to metastatic or recurrent disease, underscoring the urgent need for biomarkers that elucidate tumor behavior and refine prognosis-driven therapies. Our study addresses this unmet need systematically characterizing MRLs and establishing a novel prognostic model with clinical implications.

Migrasomes, recently identified vesicular organelles produced by migrating cells, play pivotal roles in tumor development and progression^20,44^. They carry diverse cargo, including nucleic acids, proteins, lipids, enzymes, and metabolites, offering insights into migrating cell physiology^12,19^. Detectable in blood and urine^19,45,46^, migrasomes represent a promising non-invasive source for liquid biopsy-based disease diagnosis and potential biomarkers in various physiological and pathological contexts^19,46^. These structures are also essential for cell-to-cell communication, homeostatic regulation, embryonic development, and diseases onset^47^. However, their association with GC remains underexplored, warranting further investigation to clarify this relationship.

As a newly discovered organelle, the relationship between migrasomes and tumors is gradually being uncovered. The formation of migrasome is mediated by the assembly of micro-scale tetraspanins macrodomains and the recruitment of Tetraspanin 4 (TSPAN4)^20^. Integrin β1 (ITGB1) and integrin α5 (ITGA5) have been found to exert essential functions in the formation of migrasome, exhibiting enrichment within these structures, thus implicating them as potential biomarkers for migrasomes detection^22^. N-deacetylase/N-sulfotransferase 1 (NDST1) is closely linked to migrasome, acting as both a specific marker and potential regulator, potentially playing a crucial role in cancer progression and offering insights for cancer therapy development^48^. PIGK encodes a key component of the GPI-TA complex and functions as a quality control factor within the GPIT complex^49,50^. It is essential for protein-GPI anchoring, and loss of PIGK may impair normal migrasome formation, potentially affecting cellular migration^48^. EOGT serves as a biomarker for identifying migrasomes and is involved in the assembly and functional regulation of their membrane domains. Its O-linked N-acetylglucosamine transferase activity is essential for migrasome formation^51^. Additionally, EOGT plays a role in signaling and intercellular communication within migrasomes^48^. Carboxypeptidase Q (CPQ) is the gene encoding a metallopeptidase of the M28 family, which has been shown to play a crucial function in the breakdown of circulating peptides in human plasma^52^. Previous studies have demonstrated that CPQ is enriched in migrasomes and has been established as a marker protein^19^. Research conducted by Wendy et al. elucidated that EPCIP, PDK1, and PDK2 exert crucial roles in both the formation of migrasome and the modulation of their related functions^23^. Current prognostic models for GC emphasize immune microenvironment regulation and therapeutic responses^40,41,53^, yet the role of migrasomes remains unexplored, particularly their diagnostic and therapeutic potential. While lncRNAs are well-established in GC pathogenesis^54,55^, the interplay between lncRNAs and migrasome dynamics remains unexamined. Systematic characterization of MRLs is thus imperative to define their biological significance and clinical relevance, highlighting the need for integrated studies in this emerging field. In this study, we investigated these MRLs and subsequently developed a novel risk prognostic model that leverages the expression profiles of lncRNAs, with the goal of improving the accuracy of prognostic evaluations for GC patients.

Our analysis identified 29 MRLs associated with GC risk. Among these, LINC01235, AC005165.1, AC084757.3, MIR1915HG, AP002518.2, AC037198.1, LINC00163, LINC01150, MIR4435-2HG, PGM5P4-AS1, LINC01537, AL034550.2, and AC107021.2, have been previously reported, while the remaining lncRNAs are novel discoveries in this study. LINC01235 regulates GC cell migration and invasion^56^ and metastasis^57^. AC005165.1 modulates FRZB expression in osteoarthritis^58^ and may contribute to GC progression^59^. AC084757.3 is implicated in lung adenocarcinoma via PI3K/Akt/mTOR pathway^60^. MIR1915HG influences GC development through hypoxia-related pathways^61^. AP002518.2 is associated with Wilms tumor^62^, while AC037198.1 affects GC, progression through hypoxia^61^ and angiogenesis^63^. LINC00163 is linked to gastric^64^, bladder^65^, and lung^66^ cancers, and LINC01150 is associated with gastric cancer^67^, lung^68^, and other diseases^69^. MIR4435-2HG plays a significant role in GC progression^70^,and PGM5P4-AS1 is implicated in cancer development^71^. LINC01537 may contribute to GC through disulfidptosis^72^, AL034550.2 through immune-related processes^73^, and AC107021.2 through necroptosis-related pathways^74^.

We developed a risk prognostic model incorporating four key MRLs, which effectively stratified GC patients into distinct training and test sets, demonstrating strong predictive performance for patients’ outcomes. This LncRNA-based model emerged as an independent prognostic predictor for GC. Further analysis revealed significant associations between risk scores and clinical attributes, including age and tumor grade, highlighting its clinical relevance. These findings underscore the potential of lncRNA-based models to provide personalized survival risk evaluations across GC patient subgroups, thereby informing tailored therapeutic strategies and improving patient care. Given the established role of migrasomes in tumorigenesis^20,44^and LncRNA-based risk prognostic models that have been reported in several tumors^75,76^, our method of MRLs-based prognostic model has the potential to extend to tumors more than GC. However, due to the tissue-specific nature of lncRNA, the specific LncRNA profile needs to be further established.

Functional enrichment analysis revealed significant associations between risk-associated genes and biological processes, including the cAMP^77^ and calcium signaling pathway^78^, both crucial in GC progression. Given the TME pivotal role in tumor evolution, MRLs may influence tumor progression by modulating immune regulatory pathways. Immune infiltration identified distinct TME characteristics between risk groups, with the high-risk group showing heightened immune activation and elevated tumor mutational burden, suggesting greater responsiveness to immunotherapy. These findings highlight the prognostic model’s utility in guiding immunotherapy decisions and personalized treatment strategies for GC patients.

Drug sensitivity analysis identified afatinib, gefitinib, lapatinib, oxaliplatin, and 5-FU as potential therapeutic options for high-risk GC patients. Afatinib irreversibly inhibits EGFR and ErbB2 tyrosine kinases, reducing tumor cell proliferation, invasion, and metastasis, particularly in tumors with EGFR/ErbB2 overexpression or mutations^79^. Gefitinib inhibits EGFR tyrosine kinase activity, suppressing tumor cell proliferation, invasion, and metastasis while promoting apoptosis, which is relevant given EGFR overexpression in GC^80^. Lapatinib targets both EGFR and HER2, reducing tumor cell proliferation and survival, particularly in GC with EGFR/HER2 overexpression or mutation^81^. Oxaliplatin forms DNA cross-links, disrupting replication and transcription, and has demonstrated significant anti-tumor activity in GC^82^. 5-FU inhibits thymidylate synthase and DNA replication, making it a widely used treatment for GC^83^ and other cancer.

While numerous prognosis models for GC exist, our MRLs-linked model demonstrates exceptional stability and broad applicability in subgroup analyses, offering significant potential for advancing cancer research. Beyond predictive models, we conducted comprehensive analyses to explore migrasome’s role in GC patients. However, it is essential to acknowledge several limitations of our study. First, our findings are based on publicly available data and have not yet been experimentally validated. Second, our investigation into the role of MRLs in anticancer processes remains preliminary. Lastly, while our model has undergone internal validation, it still awaits external confirmation to confirm its generalizability. These limitations outline critical areas for future research.

## Conclusions

In our study, we developed a prognostic model utilizing MRLs through comprehensive bioinformatics analyses. This risk model was leveraged to evaluate immune landscape characteristics, TMB and drug sensitivity among diverse GC patient populations. This model offers invaluable insights into clinical prognosis and therapeutic management of GC. The lncRNAs identified within the scope of this study, which are associated with migrasomes, not only enhance our understanding of GC pathogenesis but also emerge as promising therapeutic targets for this malignancy.

## Electronic supplementary material

Below is the link to the electronic supplementary material.

Supplementary Material 1

Supplementary Material 2

## Data Availability

Data is provided within the manuscript or supplementary information files.
