# Supplementary material for: A novel prognostic model based on migrasome-related LncRNAs for gastric cancer
Source: Sci Rep. 2025 Apr 25;15:14572. doi: 10.1038/s41598-025-99781-4 (PMC12032148; doi:10.1038/s41598-025-99781-4)
Supplement: Supplementary file 2 — Supplementary Material 2 [file 41598_2025_99781_MOESM2_ESM.docx]

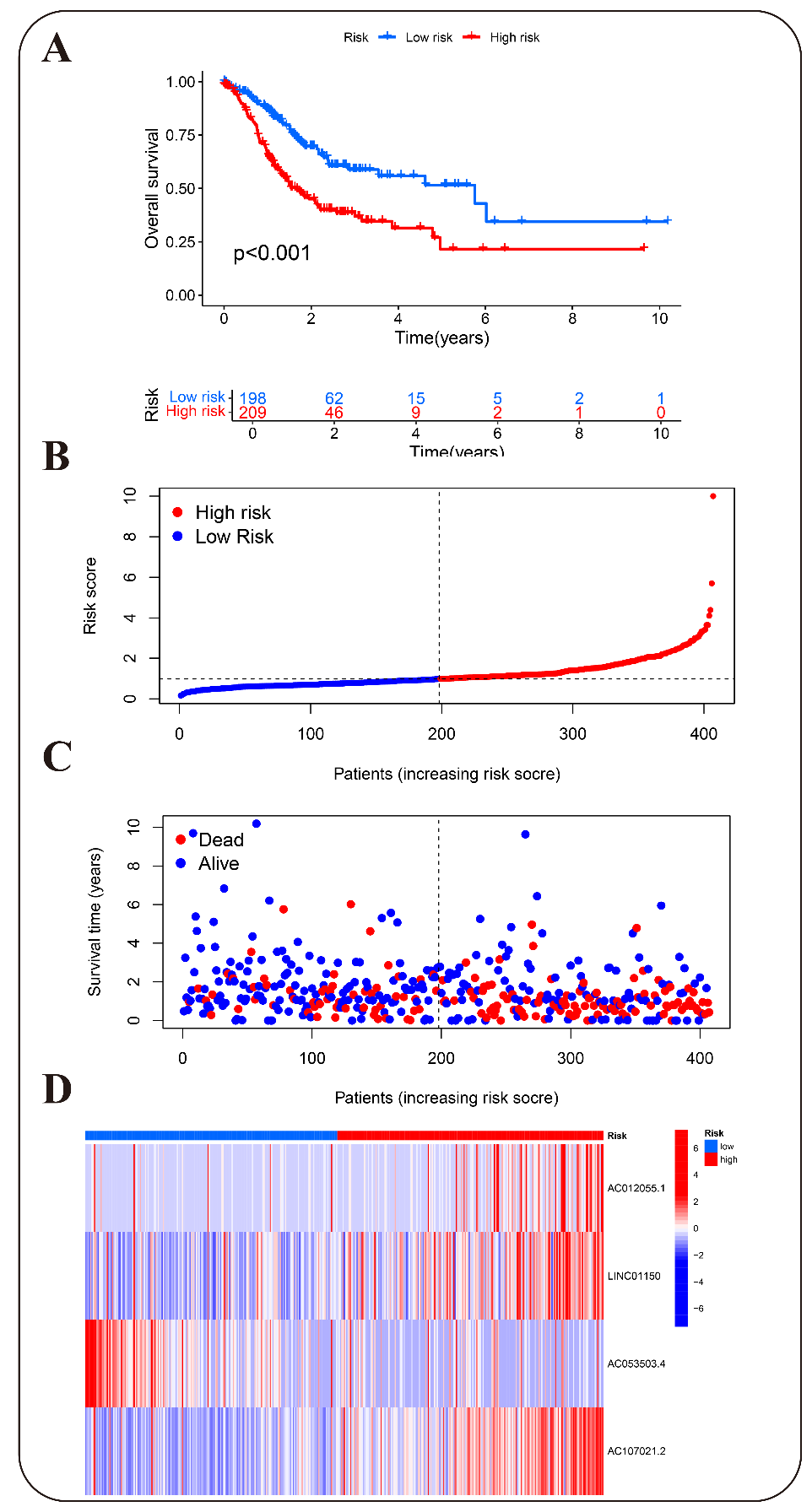


**Figure S1. Grouping and Assessment of Risk Prognostic Models.** (A) K-M survival curve for patients in the entire TCGA dataset. (B) Scatter plot representing risk scores assigned to patients in the entire TCGA dataset. (C) Scatter plot illustrating survival status of patients in the entire TCGA dataset. (D) Cluster analysis plot depicting the risk prognostic model based on four lncRNAs for patients in the entire TCGA dataset.


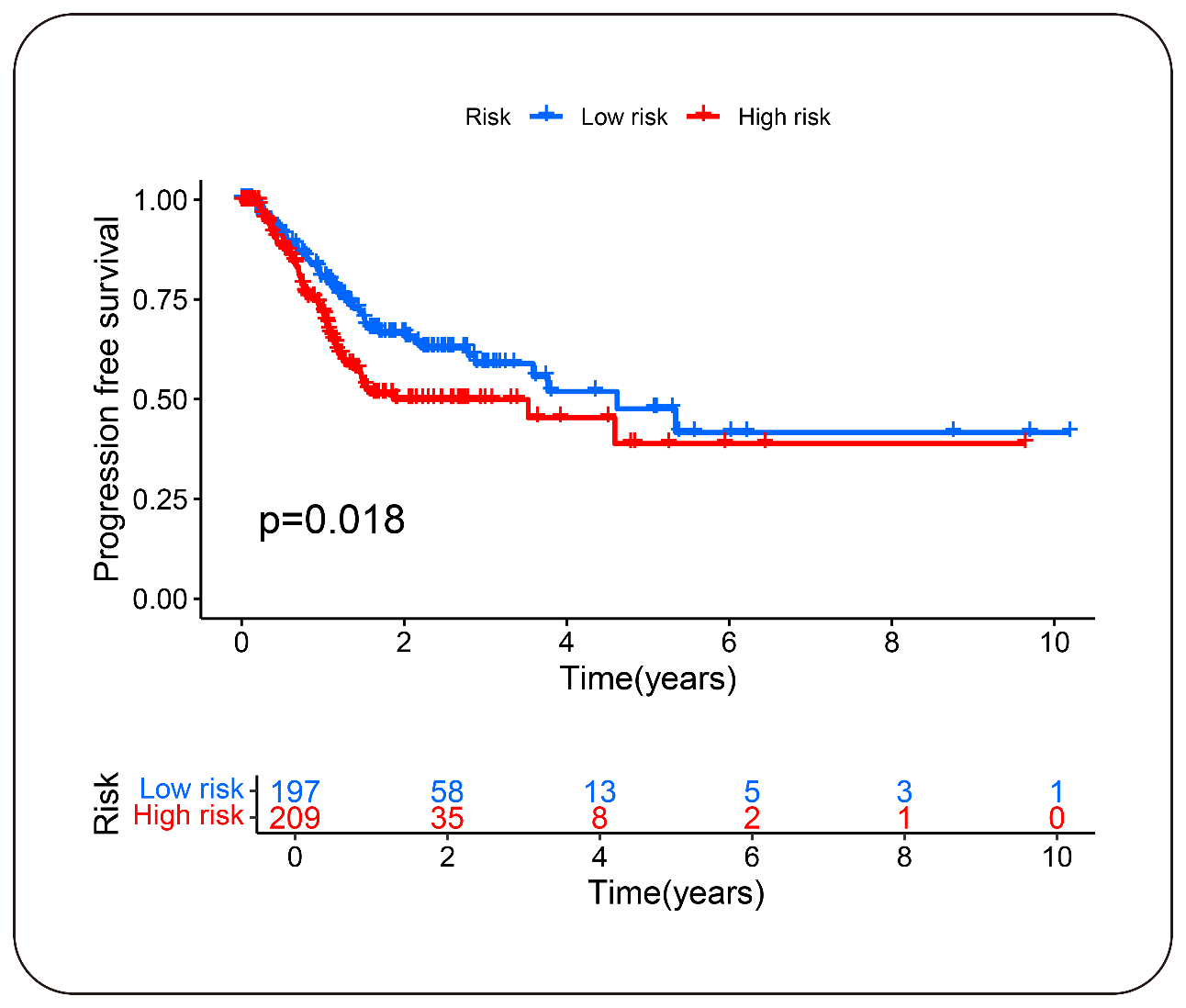


**Figure S2. Progression free survival in the entire TCGA dataset.**


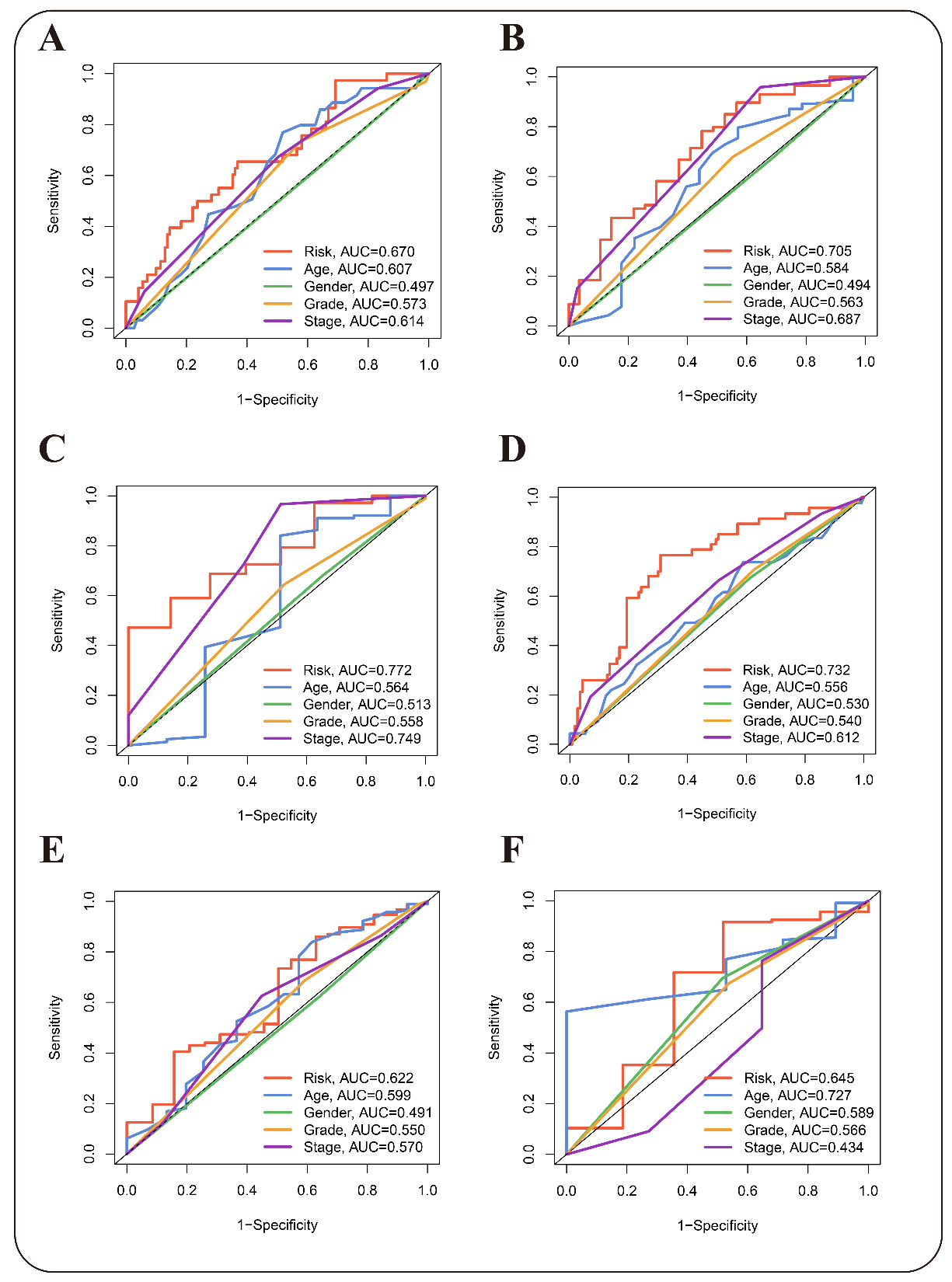


**Figure S3. The ROC results in the training and test set.** ROC curves depicting the trade-off between sensitivity and specificity for predicting 1-year (A), 3-year (B), and 5-year (C) survival across various variables in the training set, and corresponding ROC curves for predicting 1-year (D), 3-year (E), and 5-year (F) survival in the test set.


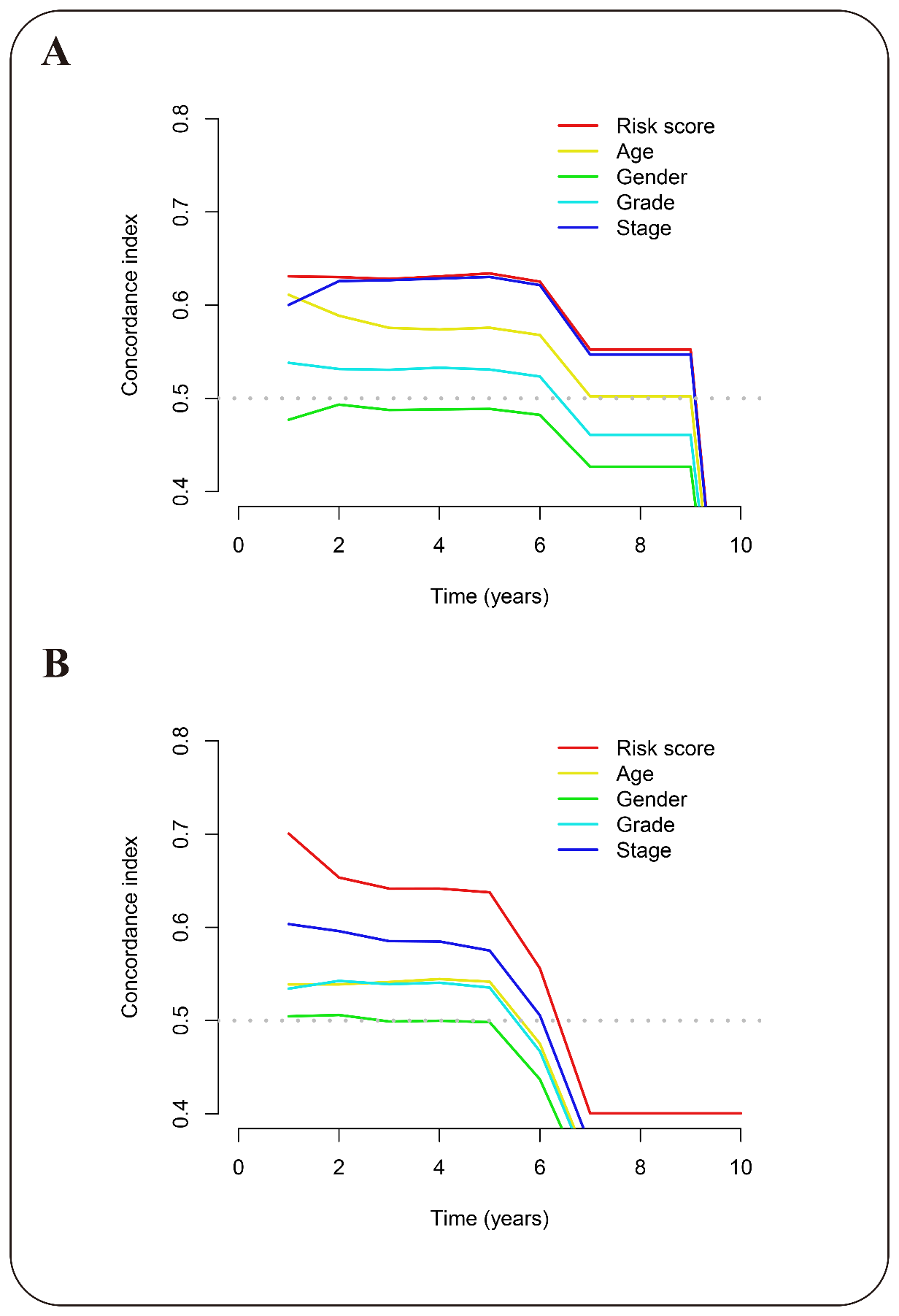


**Figure S4. The C-index Validation in the training and test set.** (A) C-index validation in the training set. (B) C-index validation in the test set.


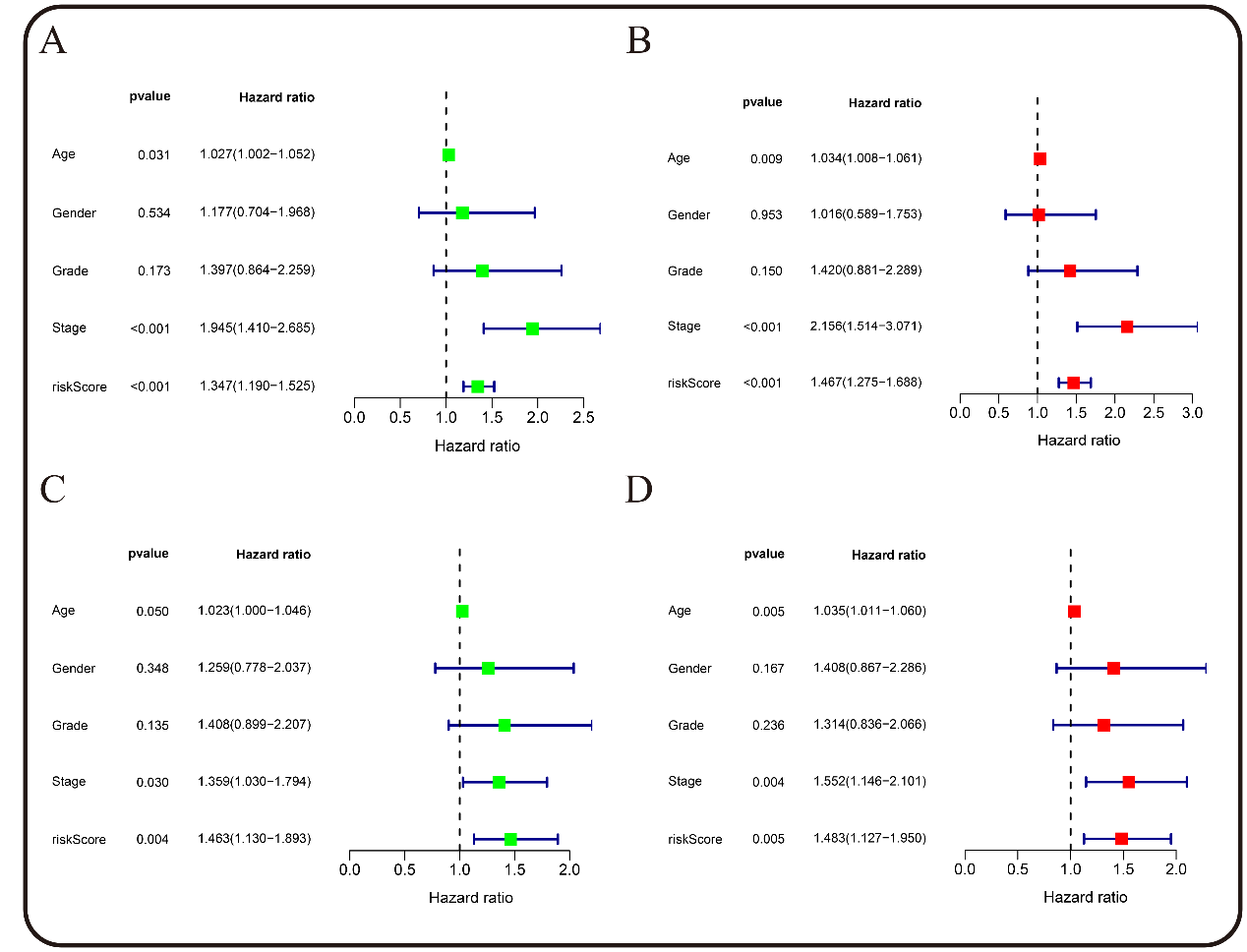


**Figure S5. Univariate and multivariate Cox regression analyses of the prognostic risk score in training and test set.** (A) Forest plot of univariate Cox regression analysis in the training set. (B) Forest plot of multivariate Cox regression analysis in the training set. (C) Forest plot of univariate Cox regression analysis in the test set. (D) Forest plot of multivariate Cox regression analysis in the test set.


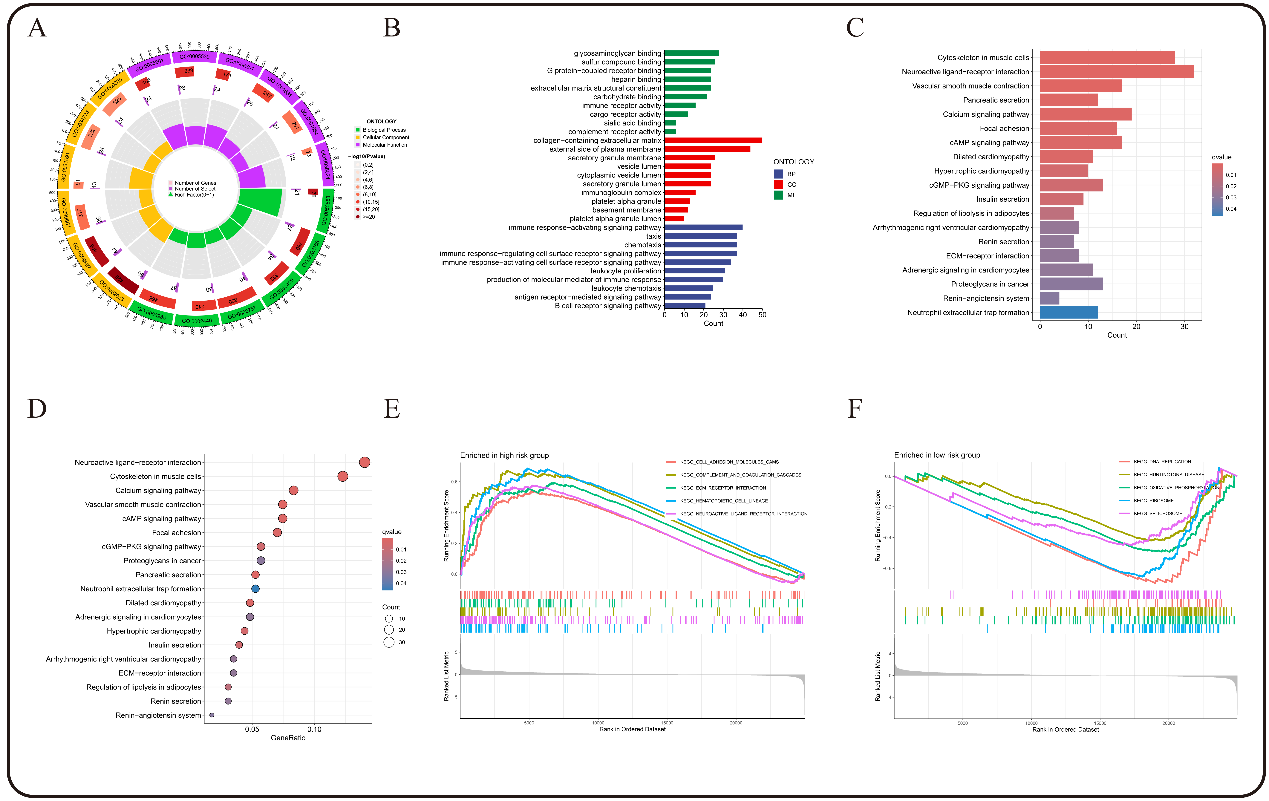


**Figure S6. Signaling Pathways Enriched in the training set.** (A) Circular graph depicting the results of GO analysis. (B) Bar graph illustrates the GO. (C) Bubble diagram representing the KEGG results. (D) Bar graph showing the KEGG results. (E) GSEA results of the high-risk group. (F) GSEA results of the low-risk group.


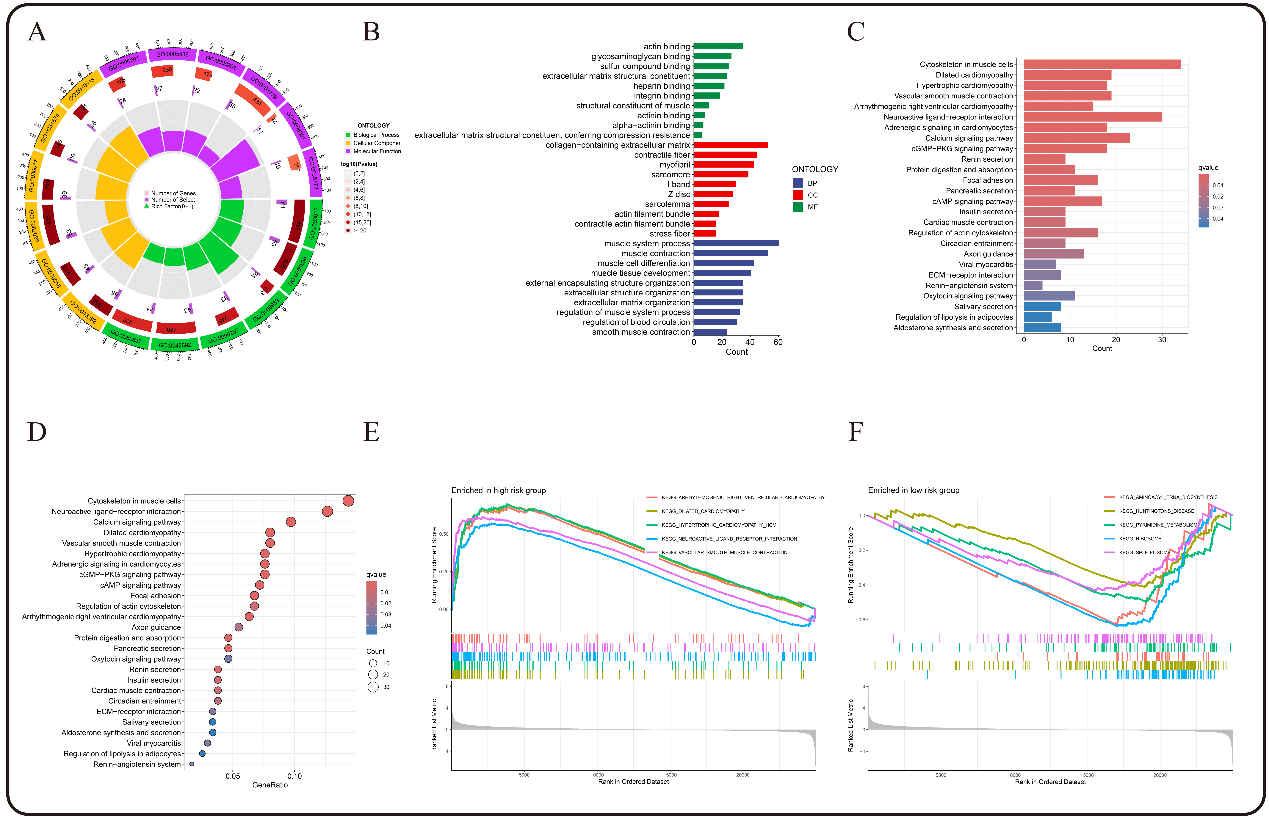


**Figure S7. Signaling Pathways Enriched in the test set.** (A) Circular graph depicting the results of GO analysis. (B) Bar graph illustrates the GO. (C) Bubble diagram representing the KEGG results. (D) Bar graph showing the KEGG results. (E) GSEA results of the high-risk group. (F) GSEA results of the low-risk group.


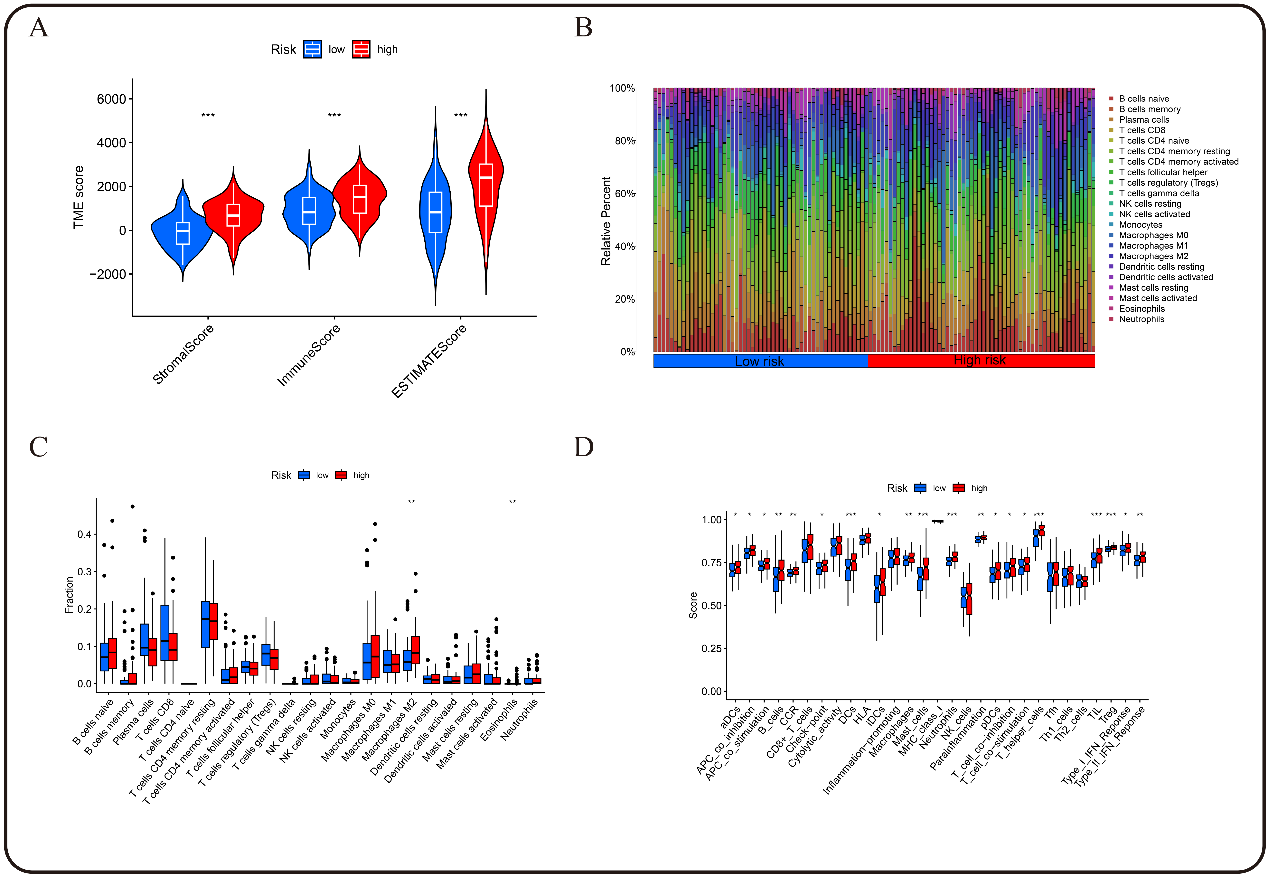


**Figure S8. Immunological Profile Analysis of the Prognostic Model in the training set.** (A) Tumor microenvironment analysis. (B) Immune cell percentage distribution. (C) Immune score analysis for both groups. (D) immunofunctional analysis. *p < 0.05, **p < 0.01, ***p < 0.001.


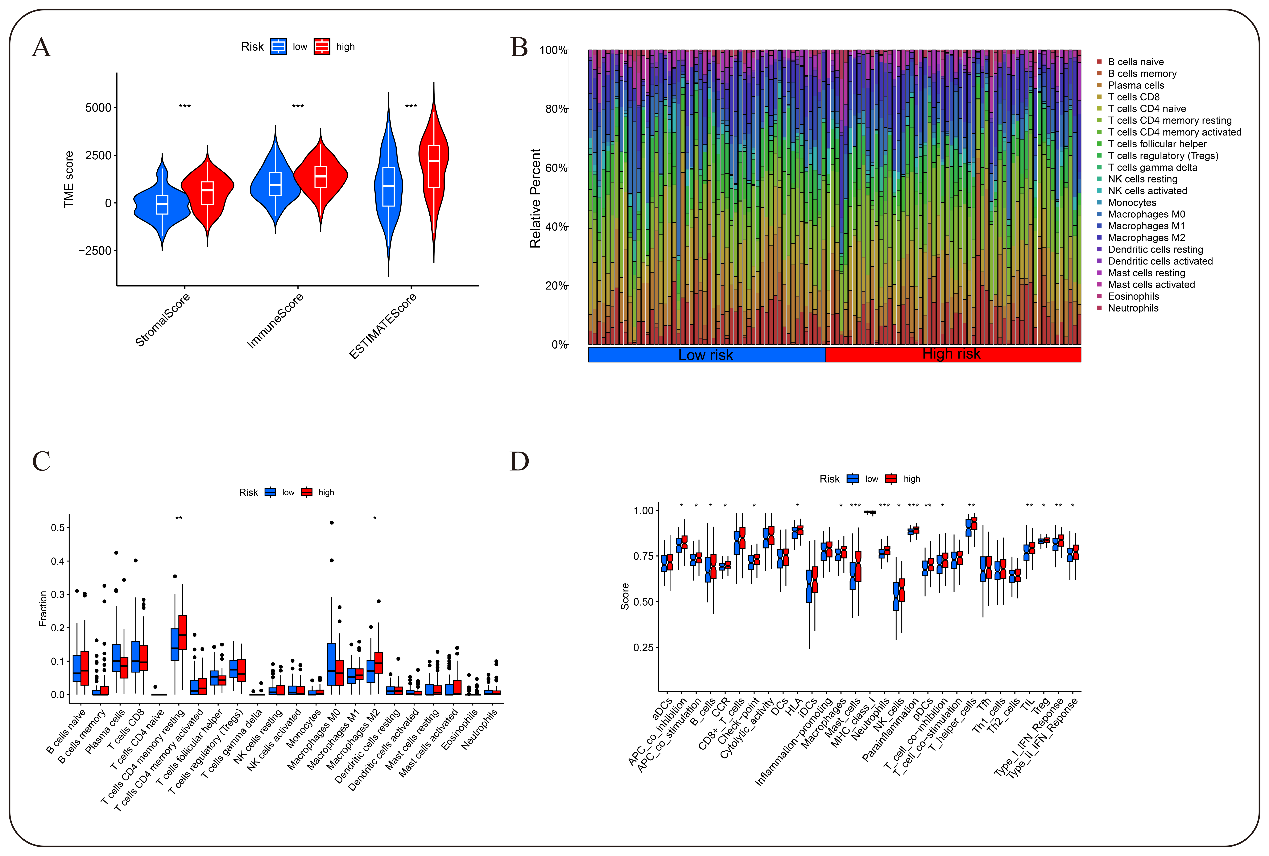


**Figure S9. Immunological Profile Analysis of the Prognostic Model in the test set.** (A) Tumor microenvironment analysis. (B) Immune cell percentage distribution. (C) Immune score analysis for both groups. (D) immunofunctional analysis. *p < 0.05, **p < 0.01, ***p < 0.001.


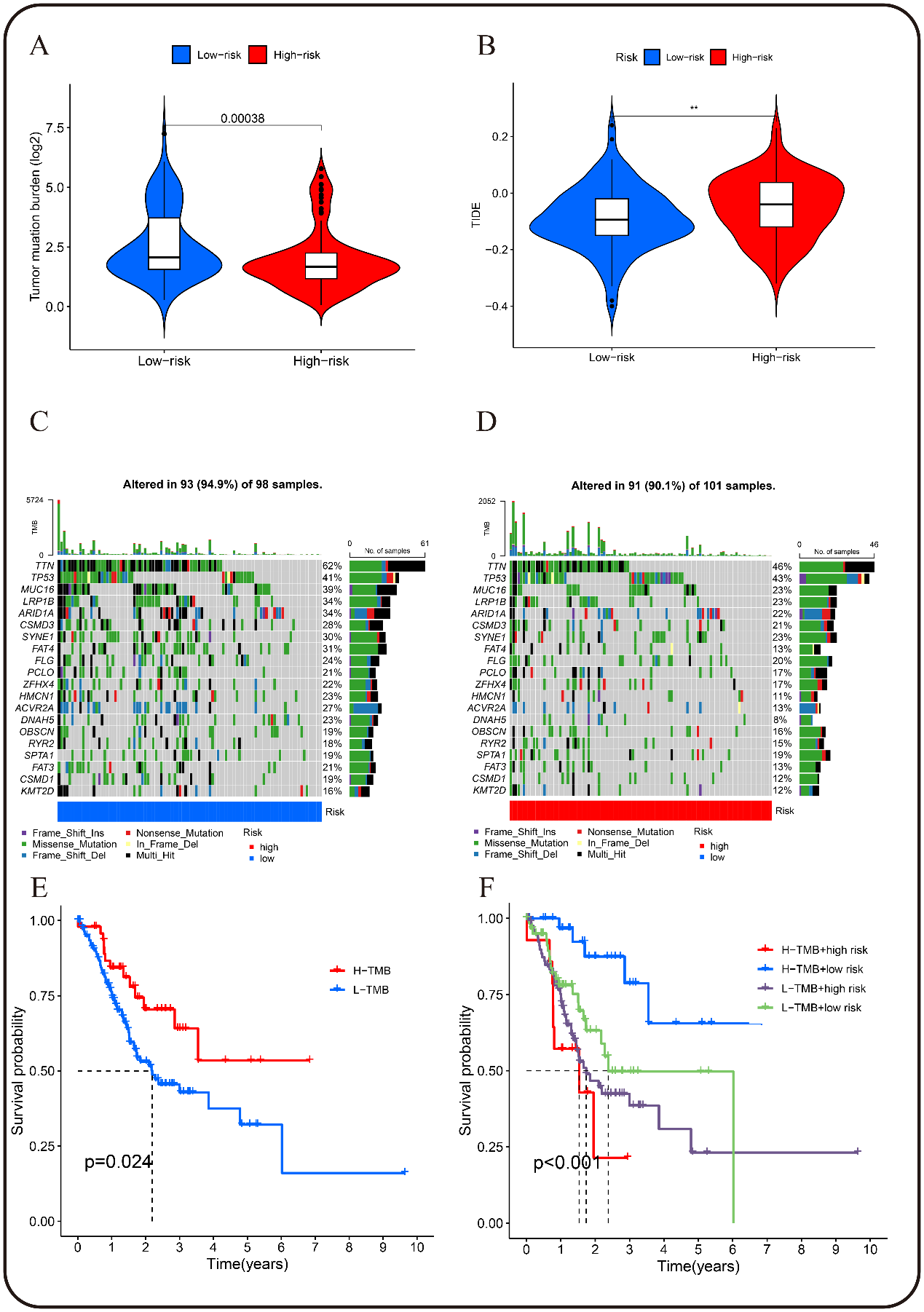


**Figure S10. Analysis of Immunosignatures in Prognostic Models within the Training set.** (A) TMB scoring. (B) TIDE analysis. (C) Waterfall plot depicting the 20 most frequently mutated genes in the low-risk subgroup. (D) Waterfall plot illustrates the 20 most frequently mutated genes in the high-risk subgroup. (E) K-M survival analysis of the two groups with high and low TMB scores. (F) K-M survival analysis of four subgroups categorized by TMB and risk status. ***p < 0.01.


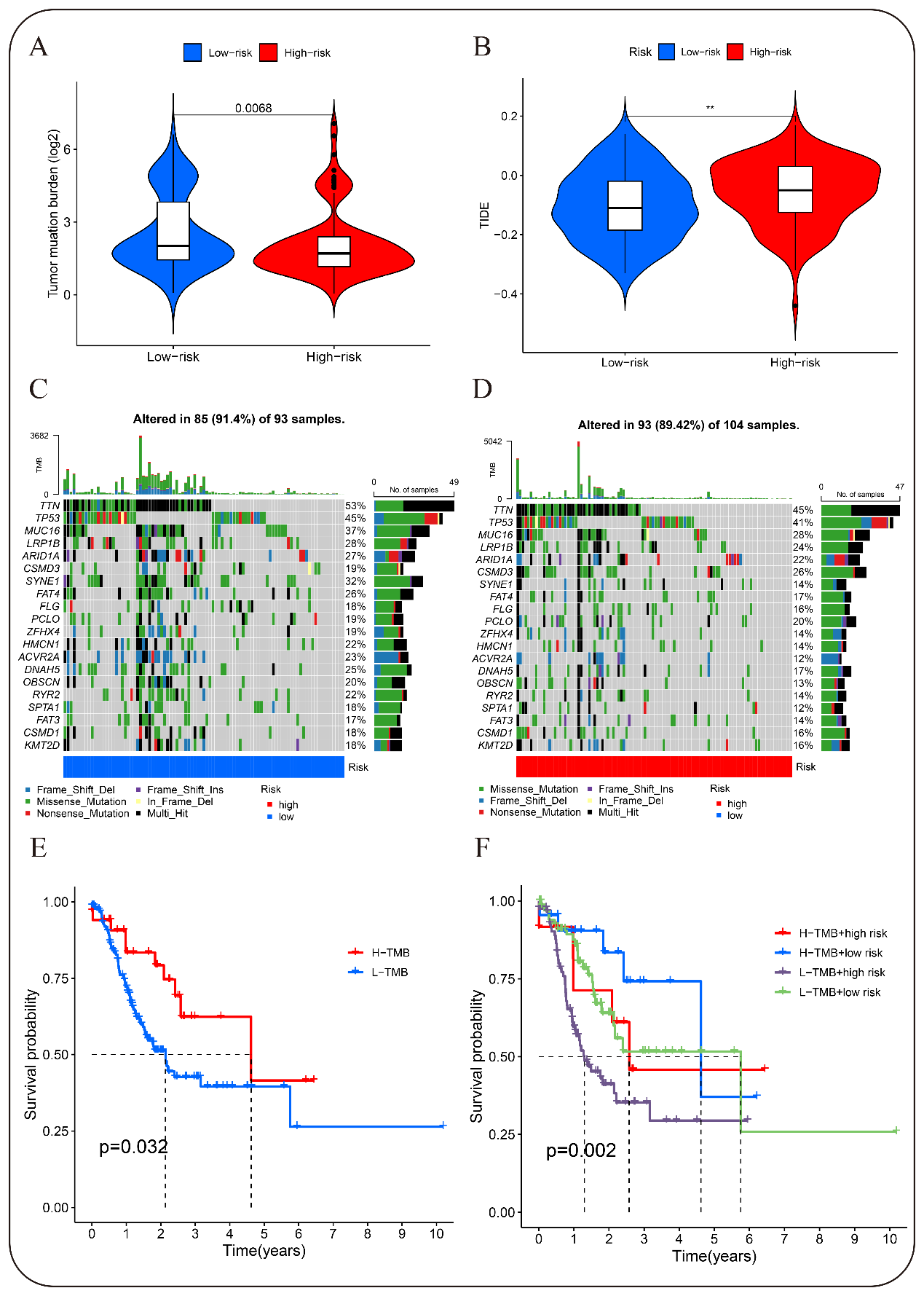


**Figure S11. Analysis of Immunosignatures in Prognostic Models within the Test set.** (A) TMB scoring. (B) TIDE analysis. (C) Waterfall plot depicting the 20 most frequently mutated genes in the low-risk subgroup. (D) Waterfall plot illustrates the 20 most frequently mutated genes in the high-risk subgroup. (E) K-M survival analysis of the two groups with high and low TMB scores. (F) K-M survival analysis of four subgroups categorized by TMB and risk status. ***p < 0.01.


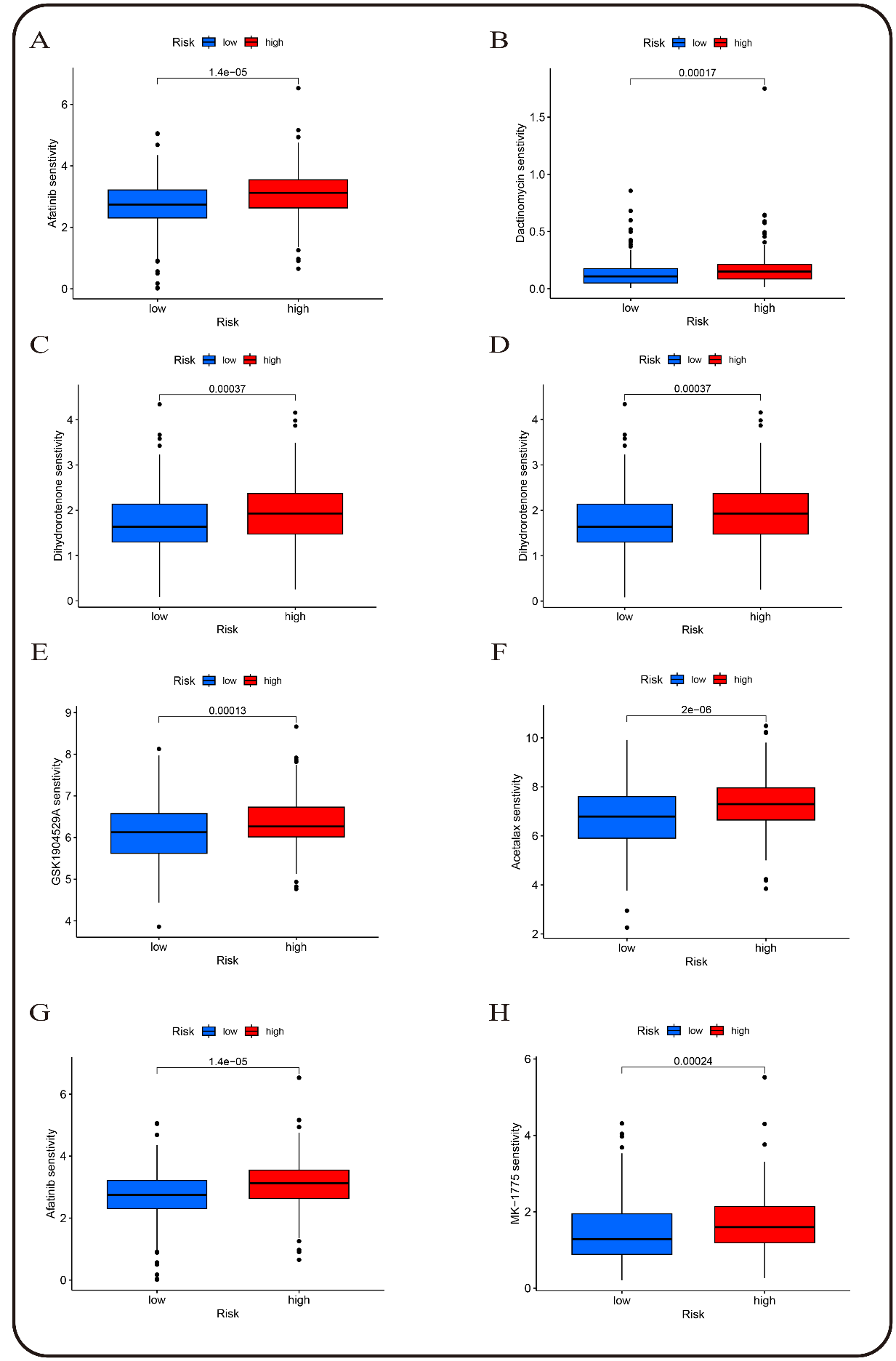


**Figure S12. Analysis of Drug Sensitivity in the Risk Prognostic Model.** *** p-value < 0.001.


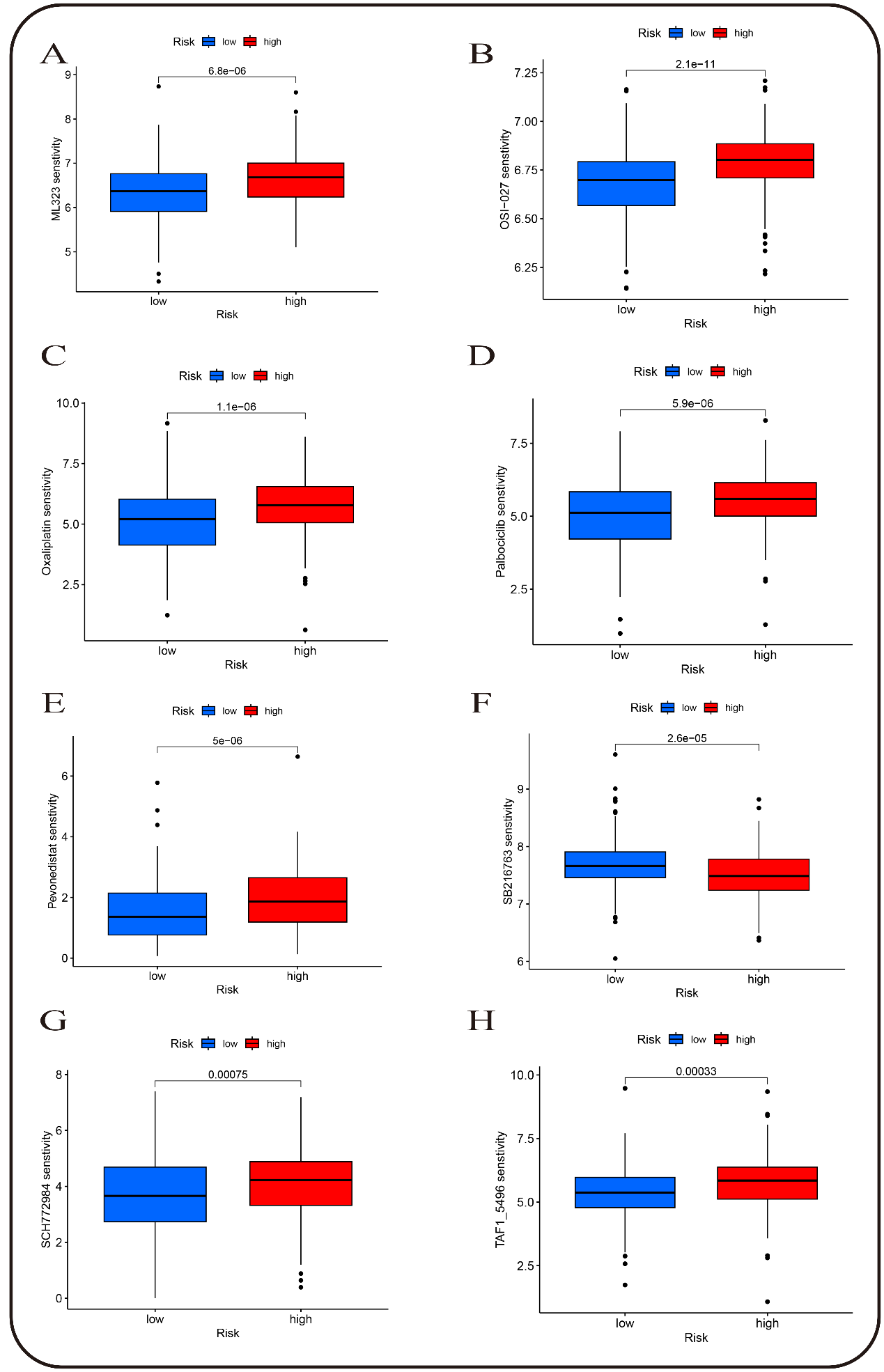


**Figure S13. Analysis of Drug Sensitivity in the Risk Prognostic Model.** *** p-value < 0.001.


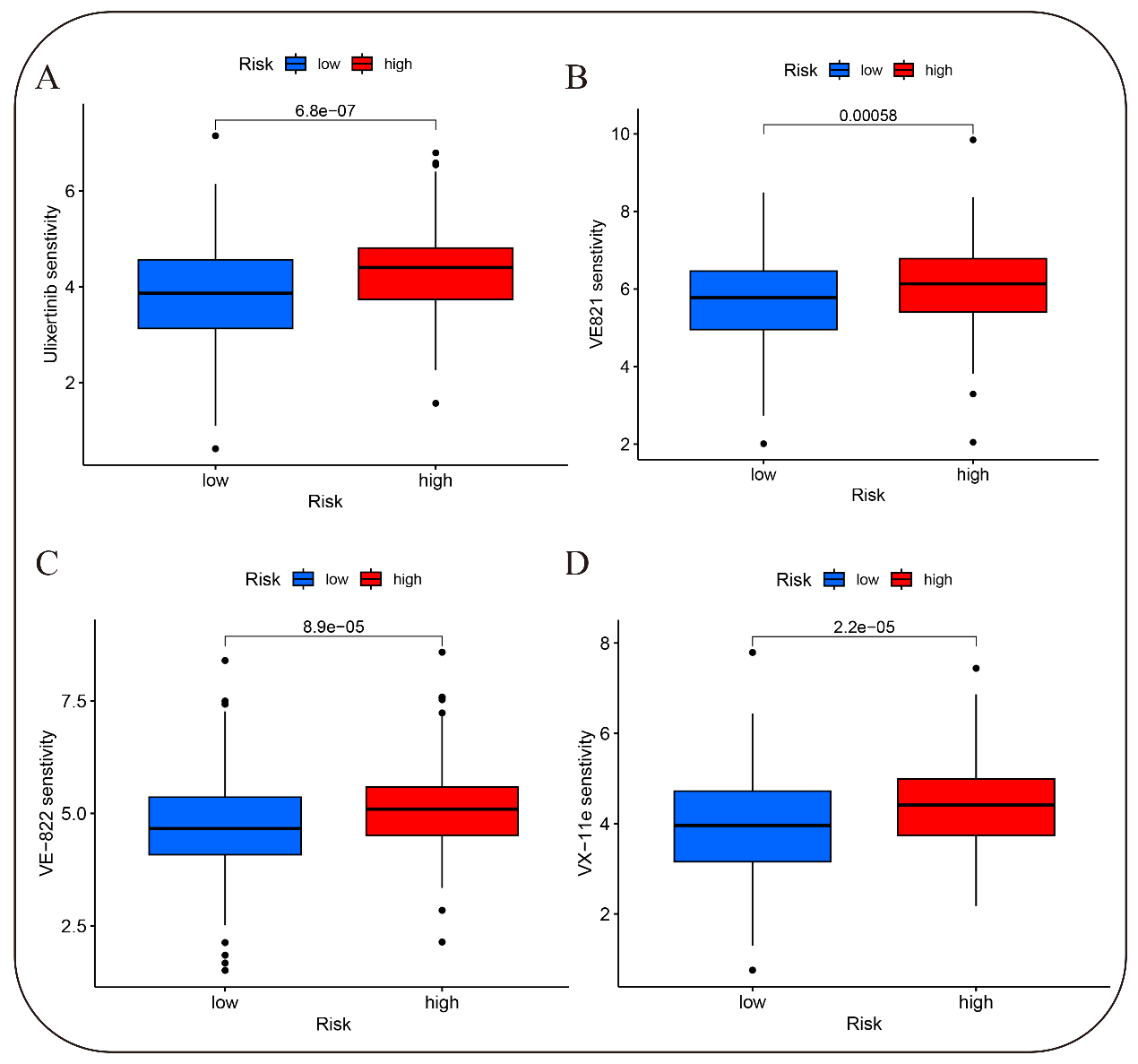


**Figure S14. Analysis of Drug Sensitivity in the Risk Prognostic Model.** *** p-value < 0.001.
